# From Guidelines to Practice: Pilot Implementation and Analytical Boundaries of a Focused ADPKD-Spectrum Gene Panel

**DOI:** 10.3390/diagnostics16172770

**Published:** 2026-08-28

**Authors:** Ramona G. Babici, Iuliu C. Ivanov, Bogdan D. Agavriloaei, Irina L. Mititiuc, Gianina Dodi, Mihaiela L. Dragoș, Stela Racoviță, Alexandra Savuca, Mugurel Apetrii, Roxana Popescu, Adrian C. Covic, Cristina Rusu

**Affiliations:** 1 Grigore T. Popa University of Medicine and Pharmacy Iasi, 700115 Iasi, Romania; tutu.ramona-geanina@d.umfiasi.ro (R.G.B.); bogdan-dumitru.agavriloaei@umfiasi.ro (B.D.A.); luanda.mititiuc@umfiasi.ro (I.L.M.); mugurel.apetrii@umfiasi.ro (M.A.); roxana.popescu@umfiasi.ro (R.P.); adrian.covic@umfiasi.ro (A.C.C.); cristina.rusu@umfiasi.ro (C.R.); 2 Dialysis and Renal Transplant Center, Nephrology Clinic, Dr. C.I. Parhon University Hospital, 700503 Iasi, Romania; 3 Molecular Diagnosis Department, Regional Institute of Oncology, 700483 Iasi, Romaniadragosloredana.z@gmail.com (M.L.D.); 4 Faculty of Biology, Alexandru Ioan Cuza University of Iasi, 700506 Iasi, Romania; 5 Nicolae Testemițanu State University of Medicine and Pharmacy, MD-2005 Chisinau, Moldova; stela.racovita@usmf.md; 6 Origyn Fertility Center, 700032 Iasi, Romania; alexandra.savuca@yahoo.com; 7 Medical Genetics Department, Saint Mary Emergency Children’s Hospital, 700309 Iasi, Romania

**Keywords:** ADPKD, *PKD1*, *HNF1B*, targeted sequencing, analytical boundaries, interval-level coverage, pseudogenes, short-read sequencing

## Abstract

**Background/Objectives**: KDIGO supports molecular testing in selected autosomal dominant polycystic kidney disease (ADPKD) scenarios and targeted next-generation sequencing panels when broader evaluation is warranted; however, gene inclusion alone establishes neither analytical completeness nor clinical reportability. **Methods**: We designed and evaluated a 28-gene ADPKD-spectrum hybrid-capture panel in a 16-sample, two-configuration pilot using 694 analytical target intervals per sample. Multiplex ligation-dependent probe amplification assessed dosage in suspected *PKD1*-related disease or *TSC2*/*PKD1* contiguous-gene deletion, and whole-exome sequencing selectively supported short-read reidentification. **Results**: In the primary eight-sample dataset, 4592/5552 observations (82.7%) had interval mean depth ≥20×, 4256/5552 (76.7%) had interval minimum depth ≥20×, and 89.6% of interval-record bases reached ≥10×. Nine genes *(FLCN, GANAB, HNF1B, PRKCSH, REN, SEC61A1, TSC2, UMOD* and *VHL)* met mean depth ≥20× throughout; six also met the minimum-depth criterion. For *PKD1*, 45/46 intervals met both criteria in all samples, but exon 1 remained undercovered, and nominal depth in duplicated sequence did not establish authentic-locus callability. Signals were observed at all four positive-comparator loci, although two lacked sufficient support for independent reporting. Seven of nine panel-first cases yielded observations: one *HNF1B* and one unique-locus *PKD1* finding were supported, whereas four duplicated-sequence *PKD1* findings remained confirmation-dependent. **Conclusions**: Responsible implementation requires explicit analytical boundaries and staged complementary testing.

## 1. Introduction

ADPKD is a common monogenic cause of kidney failure and a major indication for kidney replacement therapy worldwide. It is a multisystem disorder characterized by progressive bilateral kidney cyst formation, frequent liver cysts, and variable extrarenal manifestations. In typical familial cases, diagnosis is often clinical; however, the 2025 KDIGO guideline identifies situations in which genetic testing can clarify diagnosis or prognosis, including equivocal or atypical imaging, limited cyst burden, discordance between kidney structure and function, negative or unknown family history, very-early-onset disease, evaluation of young related living donors, and reproductive planning [1].

Although pathogenic variants in *PKD1* and *PKD2* account for most genetically resolved ADPKD, contemporary testing increasingly considers a wider cystic kidney and liver disease spectrum. Monoallelic pathogenic variants in *GANAB*, *ALG9*, *DNAJB11* and *ALG5* have been associated with atypical polycystic kidney and/or liver phenotypes, whereas the interpretation of other candidate genes depends on gene–disease validity, mode of inheritance and phenotypic fit [1,2,3,4,5].

Thus, several inherited disorders should be considered in the differential diagnosis of ADPKD-like cystic kidney disease. These include *HNF1B*-related kidney disease, *COL4A3*-*COL4A5*-associated nephropathies, tuberous sclerosis complex, and *TSC2*/*PKD1* contiguous-gene deletion syndrome [6,7,8,9]. Other differentials for cystic or tumor-predisposition phenotypes with distinct surveillance and counseling implications include von Hippel–Lindau disease and Birt–Hogg–Dubé syndrome [10,11].

More recent outcome data are consistent with this broader diagnostic framework. The highest pooled diagnostic yield in cystic kidney disease was observed in a systematic review on testing in adult CKD, but the yield was dependent on selection and testing strategy [12]. Multiple findings in the same patient, biallelic or digenic configurations and mosaicism have also been reported in ADPKD-specific panel cohorts [13]. Independent ADPKD cohort data have likewise demonstrated the value of molecular testing for diagnostic verification and for identifying overlapping phenotypes that can mimic ADPKD [14]. In broader cystic kidney disease referral cohorts, multigene panel testing has also highlighted the clinical relevance of copy-number variant detection, supporting variant-class-aware assay design and interpretation [15].

KDIGO recommends targeted multigene testing as a pragmatic approach when molecular testing is indicated in clinically selected scenarios [1]. Targeted long-read sequencing can resolve *PKD1* variants and structural events that remain difficult to detect after ordinary short-read testing, including pseudogene-related events, high-GC exon 1, indels, deep intronic variants and copy-number changes [16]. Nevertheless, targeted short-read hybrid-capture panels still have a place within the framework of existing instrumentation and laboratory workflows, provided their limitations and requirements for complementary testing are clearly spelled out [17,18].

However, gene content alone is not sufficient to assess a panel’s utility for routine implementation. Standards for diagnostic panels include gene–disease validity, transcript selection, technical completeness of the assay, homologous-sequence interference, the scope of copy-number variation, and clarity of reporting limitations [17]. Moreover, NGS standards for constitutional testing require method validation, continuous quality monitoring, and assay-specific reporting [19]. These principles are especially relevant to ADPKD because short-read assays are susceptible to *PKD1* pseudogene homology, focal low-depth or high-GC regions, and pipeline-dependent structural-variant detection [16,20,21].

Against this background, we evaluated a focused 28-gene ADPKD-spectrum panel designed for classical ADPKD and selected overlapping hereditary kidney disorders as a candidate first component of a staged genetic testing pathway pending formal analytical validation. The panel was intentionally limited to genes with the greatest expected relevance in our referral setting and was not designed to capture the full KDIGO differential diagnosis or all minor ADPKD-associated genes. Sixteen samples were processed in two independent pilot configurations with distinct analytical roles. Using a fixed denominator of 694 analytical target intervals, we assessed run-specific quality control, interval- and gene-level depth completeness, the recurrent *PKD1* exon 1 limitation, authentic-locus attribution across *PKD1* regions, SNV/indel interpretation, and exploratory read-depth behavior in pre-characterized CNV reference cases. The aim was not to estimate diagnostic yield or claim full analytical validation, but to define a transparent, assay-specific framework for interpretation, reporting, and complementary testing.

## 2. Materials and Methods

### 2.1. Study Design, Sample Roles, and Ethics

**Figure 1:**
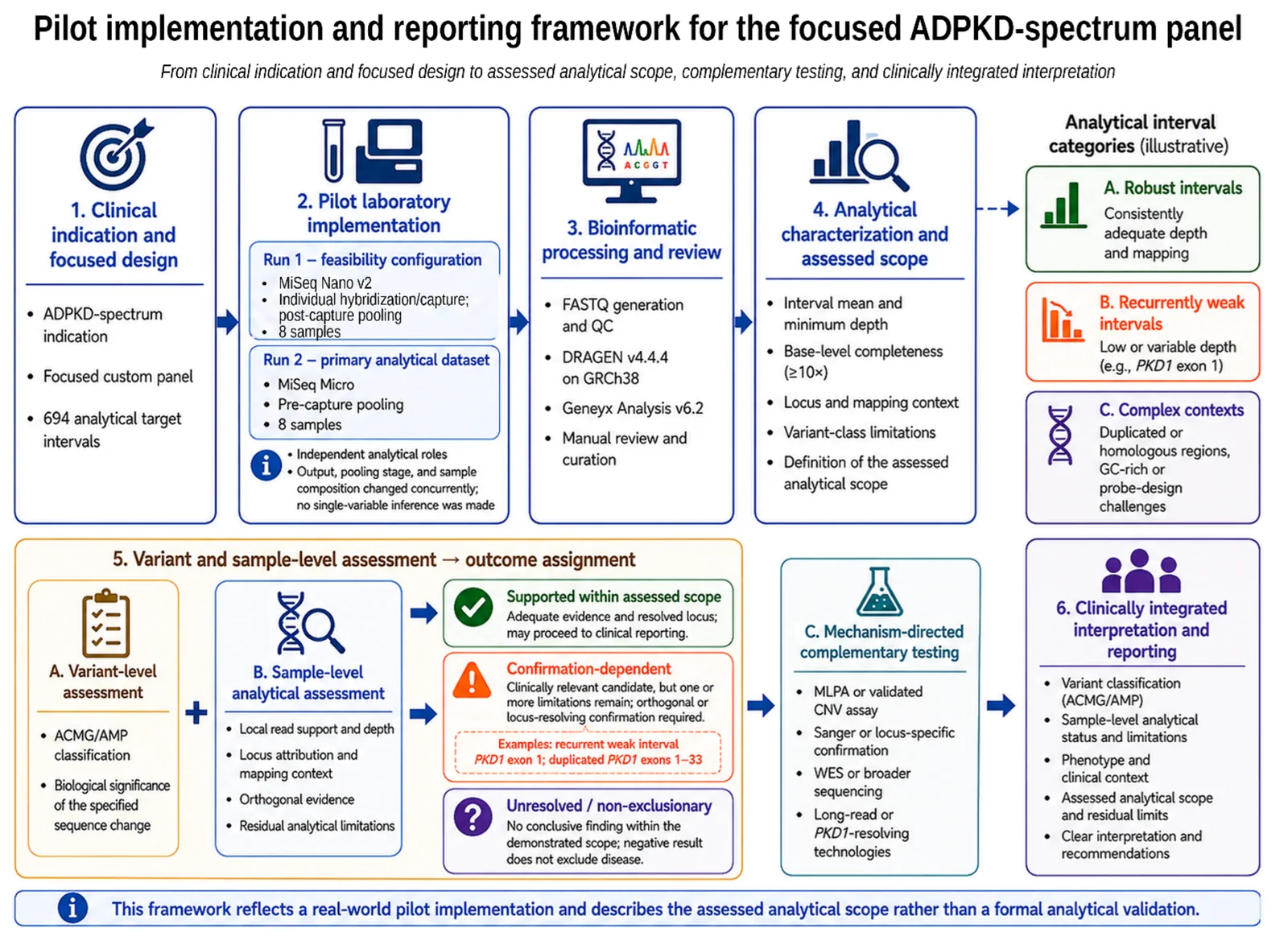
Pilot implementation, analytical assessment, and reporting framework for a focused ADPKD-spectrum panel. The framework links laboratory configuration, interval-level characterization, variant-level classification, sample-level analytical confidence, complementary testing, and clinical reporting. Run 1 was the initial low-output feasibility configuration; Run 2 was the primary higher-output dataset. Because output, pooling stage, and sample composition changed concurrently, no single-variable inference was made. Findings were categorized as supported within the assessed scope, confirmation-dependent, or unresolved/non-exclusionary. Abbreviations: ACMG/AMP, American College of Medical Genetics and Genomics/Association for Molecular Pathology; ADPKD, autosomal dominant polycystic kidney disease; CNV, copy-number variant; GRCh38, Genome Reference Consortium Human Build 38; MLPA, multiplex ligation-dependent probe amplification; QC, quality control; WES, whole-exome sequencing.

This pilot implementation study evaluated a focused ADPKD-spectrum panel within the local laboratory workflow. Sixteen samples were processed in two batches of eight. Run 1 (C1–C8) was the initial low-output feasibility configuration, whereas Run 2 (C9–C16) provided the primary higher-output analytical dataset. Sequencing output, pooling stage, and sample composition varied simultaneously; therefore, the runs were assigned different analytical roles and were not considered a controlled comparison (Figure 1).

Eligible samples were selected to represent the clinical spectrum addressed by the panel, including suspected ADPKD, atypical or unexplained hereditary cystic kidney disease, and other inherited kidney disorders covered by the design. For analysis, samples were assigned to four analytical roles: nine panel-first clinical cases, two pre-NGS CNV reference cases, four previously characterized sequence-variant comparators, and one previously characterized negative comparator (Table 1). Reference and comparator samples were used for concordance and analytical boundary assessment and were not counted as de novo panel-first findings. One comparator case, C13, had been published previously [22].

Ethics approval was obtained from the Ethics Committee of Grigore T. Popa University of Medicine and Pharmacy, Iași, Romania (approval nos. 394/01.02.2024 and 596/24.04.2025), Dr. C. I. Parhon Hospital, Iași (no. 11271/13.12.2024), and Saint Mary Emergency Children’s Hospital, Romania (no. 38065/18.12.2023). All participants, or their legal representatives where applicable, provided informed consent for genetic testing and for the scientific use of anonymized clinical and molecular data.

### 2.2. Panel Design and Fixed Analytical Target Set

The assay was evaluated as a candidate-focused panel for incorporation into a staged ADPKD-spectrum testing pathway. Gene selection was based on expected clinical relevance in the referral setting, published gene–disease associations, and phenotypic overlap with ADPKD-like hereditary kidney disorders. The prespecified study scope comprised 28 genes: *PKD1*, *PKD2*, *DNAJB11*, *GANAB*, *HNF1B*, *UMOD*, *TSC2*, *PRKCSH*, *ALG5*, *ALG8*, *ALG9*, *PKHD1*, *DZIP1L*, *TSC1*, *COL4A1*, *REN*, *SEC61A1*, *SEC63*, *LRP5*, *SEC61B*, *VHL*, *COL4A3*, *COL4A4*, *COL4A5*, *MUC1*, *FLCN*, *NOTCH2*, and *OFD1*. *PTEN* intervals were present in the vendor design/export but were outside the prespecified study scope and were excluded from the study-specific target file and all denominator-based analyses.

The panel was structured around three clinically relevant groups: major ADPKD genes, selected minor ADPKD-associated or polycystic liver disease genes, and phenocopy or overlapping hereditary kidney disease genes. Its scope was intentionally focused. The design was not intended to cover the full KDIGO differential diagnosis, all renal ciliopathy genes, or every minor ADPKD-associated gene.

Target architecture was defined gene by gene according to clinical relevance, technical feasibility, and expected diagnostic contribution. The Agilent SureDesign Entire Transcribed Region option was requested for *PKD1*, *PKD2*, *TSC1*, and *TSC2*; within the selected transcript models, this comprised coding and non-coding exons, introns, and 5′/3′ untranslated regions. Within the pseudogene-homologous portion of *PKD1*, probe selection uniqueness constraints were deliberately relaxed for selected intervals that would otherwise have been omitted. For *DNAJB11*, *HNF1B*, and *UMOD*, the request included exons, untranslated regions, and 10-bp intronic flanks; most remaining genes were represented by coding exons with 10-bp intronic flanks. Additional *HNF1B* flanking probes supported contextual exon-level dosage review. Inclusion in the capture request did not establish base-by-base analytical callability.

**Figure A1:**
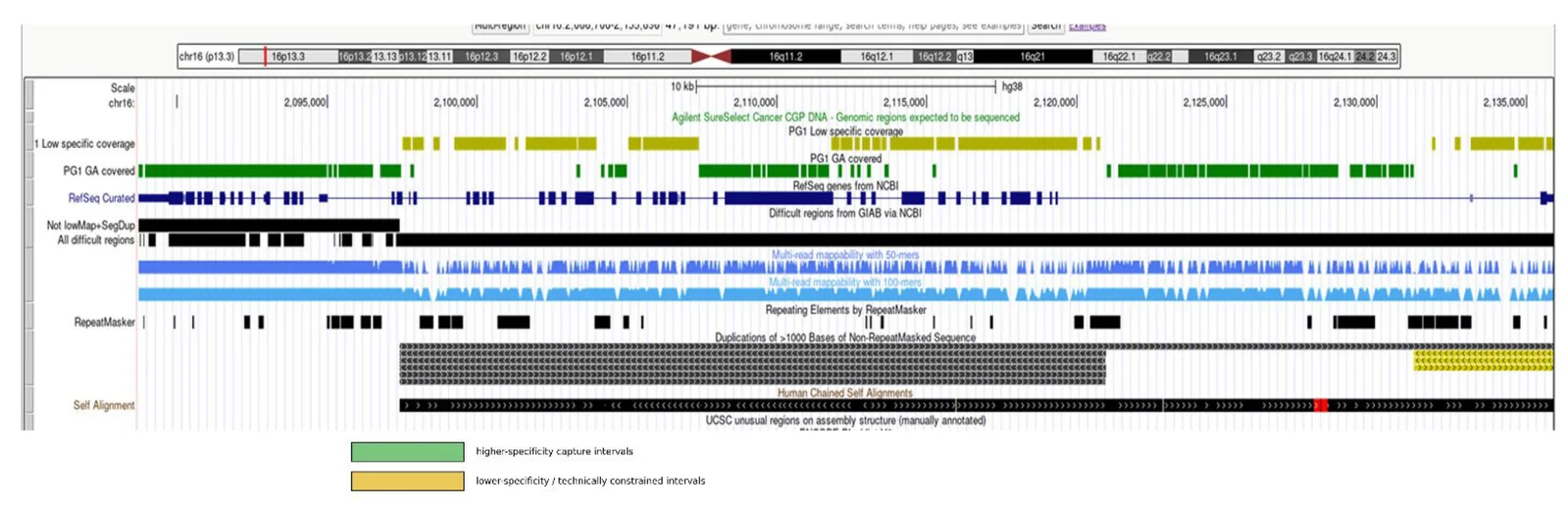
Design-stage *PKD1* targetability and probe specificity in the custom panel. The UCSC Genome Browser view shows higher- and lower-specificity probe intervals across *PKD1*. These annotations provide qualitative design context and do not establish empirical mappability or authentic-locus callability.

Probe design was performed with the Agilent SureSelect DNA Advanced Design Wizard/SureDesign platform using GRCh38/hg38. After exclusion of *PTEN*, which was outside the prespecified 28-gene study scope, the non-redundant merged genomic union of the final study-specific BED file was 339,922 bp (0-based, half-open coordinates). The BED file is provided as Supplementary Data File S1. Higher- and lower-specificity probe design tracks, particularly the *PKD1* intervals, are provided as Supplementary Data Files S2 and S3 and illustrated in Appendix A, Figure A1. The fixed 694-record analytical denominator and complete sample-by-interval matrices are provided in Supplementary Data Files S4 and S5.

### 2.3. DNA Extraction, Library Preparation, and Sequencing

Genomic DNA was extracted from EDTA-anticoagulated peripheral blood using the Wizard Genomic DNA Purification Kit (Promega, Madison, WI, USA). DNA was quantified fluorometrically with a Qubit dsDNA high-sensitivity assay, and 10–200 ng was used per library according to available material.

Both runs used the same Agilent SureSelect XT HS2 DNA chemistry and custom biotinylated RNA probe design. Core reagents included the SureSelect Enzymatic Fragmentation Kit (5191-4079), SureSelect Library Prep (5500-0146), Index Primer Pairs (5191-5687), custom RNA probes (5191-6900), Hybridization and Wash Module (5190-9685), and Post-Capture PCR Module (5191-6686), together with AMPure XP beads and Dynabeads MyOne Streptavidin T1. The workflow comprised enzymatic fragmentation, end repair/A-tailing, adaptor ligation, bead purification, indexed pre-capture amplification, probe hybridization, streptavidin-bead capture, stringent washing, post-capture amplification, and final purification. Fragmentation was performed at 37 °C for 10 min and then inactivated at 65 °C for 5 min to generate genomic inserts of approximately 180–250 bp. After adaptor ligation and amplification, the observed library molecule size distributions were approximately 300–450 bp. Library quantity and size distribution were assessed by fluorometry and capillary electrophoresis. Target-enriched libraries were sequenced on an Illumina MiSeq using paired-end 2 × 150-bp reads.

In Run 1, libraries underwent individual hybridization and capture and were pooled after post-capture amplification and quality control. The final pool was sequenced with a MiSeq Reagent Nano Kit v2 (300 cycles). In Run 2, indexed libraries were quantified and normalized, pooled before hybridization, captured as a pool, and sequenced with a MiSeq Reagent Micro Kit v2 (300 cycles).

The two runs were analyzed as independent pilot configurations with distinct analytical roles; no causal or single-variable effect was inferred from their differences. The common laboratory backbone and different pooling stages are shown in Appendix A, Figure A5.

### 2.4. Bioinformatic Processing, Quality Control, and Manual Variant Review

Following MiSeq image analysis, base calling, quality-score assignment, and index-based demultiplexing, paired-end FASTQ files were processed locally with Illumina DRAGEN v4.4.4 against GRCh38. DRAGEN generated the alignments, SNV/small-indel calls, target-coverage reports, and SV-oriented outputs used for screening-level review.

Tertiary analysis was performed in Geneyx Analysis using Annotation Version Set v6.2. Geneyx supported transcript-level annotation, configurable filtering, population-frequency and ClinVar review, phenotype-informed prioritization, gene–disease assessment, evidence retrieval, and report preparation. Principal resources used for variant interpretation included MANE, gnomAD, ClinVar, ClinGen, OMIM, MasterMind, CADD, REVEL, and SpliceAI; the complete version-set list is provided in Supplementary Methods. Candidate variants were visually inspected in the Geneyx Genome Browser viewer and in IGV using hg38 to review the read pile-up and the local genomic, transcript, and annotation context. Software-generated filters and ACMG/AMP suggestions were treated as decision support and were manually reviewed before final classification.

Global quality-control descriptors included total reads, Q30 bases, mapped and properly paired reads, duplicate and multi-mapping fractions, mapping-quality distribution, and insert-size summaries. These metrics were analyzed separately from interval-specific depth and locus-specific callability.

### 2.5. Coverage Metrics and Analytical Assessment

Coverage analyses were based on 16 DRAGEN FullCoverage_GenePanel outputs generated during the study. Eleven *PTEN* records outside the final 28-gene study scope were excluded from each 705-record export, leaving a fixed denominator of 694 analytical target-interval records per sample. Their row-wise summed length was 103,075 bp, and their merged non-redundant genomic union was 102,042 bp. Records provided interval mean, minimum, and maximum depth, as well as the number of bases below 10×. Length-weighted mean interval depth was calculated as Σ(mean depth × interval length)/Σ(interval length). For each sample, we calculated the proportions of records with mean depth ≥10×, ≥20×, and ≥30×; minimum depth ≥10× and ≥20×; and the proportion of interval-record bases covered at ≥10×.

The ≥20× interval-mean threshold was used as a descriptive benchmark, not as a universal site-level detection or reporting cutoff. Candidates in sub-benchmark intervals remained eligible for site-level review using actual DP, allelic balance, read quality, local mapping, variant class, and locus specificity; the absence of a candidate in a low-coverage or technically constrained interval was considered non-exclusionary. Negative findings were interpreted only within the demonstrated interval-, locus-, and variant-class scope relevant to the clinical question [18,19].

### 2.6. Site-Specific Locus-Aware Review of Duplicated PKD1 Sequence

Routine alignments to the complete GRCh38 reference, including *PKD1P1*–*PKD1P6*, were manually reviewed for candidate *PKD1* observations using the locus-aware approach described above. Paired FASTQ files for C2, C4, C10, and C11 were also reassessed retrospectively using a version-controlled workflow provided as Supplementary Code S1 (v1.0.0). The analysis was executed in a Docker environment using Python 3.12.13, edlib v1.3.9.post1, and mappy/minimap2 v2.30. A check-summed competitive FASTA containing authentic *PKD1* and *PKD1P1–PKD1P6* was constructed from the GRCh38 primary assembly reference using the supplied locus manifest and reference-builder script. Exact 11-mer seeds spanning the candidate REF or ALT allele were used only to retrieve candidate read pairs. Each candidate pair was then compared with locus-specific REF-like and ALT-like haplotypes for all seven loci. The best score across both allele states was retained at each locus so that the candidate event itself could not determine locus assignment. Exact edit-distance comparisons used edlib, whereas competitive mapping summaries used mappy, the Python binding to minimap2 [23,24]. A fragment contributed to the conservative locus-restricted counts only when the candidate base or indel flanks had Phred quality ≥30, non-candidate paralogous sequence variants excluded all compatible pseudogene competitors represented at that site, authentic *PKD1* was the unique best-scoring locus with an edit-distance margin ≥2, and the paired reads showed coherent inward-facing geometry. These counts used a different recovery denominator from nominal DRAGEN DP and were therefore not compared directly.

This retrospective, site-specific analysis provided reproducible computational research evidence but was not a prospectively validated *PKD1*-resolving clinical assay. It did not establish global callability across duplicated *PKD1* exons 1–33 and did not replace a validated locus-specific or long-read clinical method [20,25,26,27]. The source code, version-controlled Docker environment, software requirements, reference-builder script, locus manifest, checksums, and example output summaries are provided in Supplementary Code S1 (v1.0.0).

### 2.7. Variant Classification, Sample-Level Analytical Status and Complementary Testing

Candidate SNVs/small indels were classified within the ACMG/AMP five-tier framework. Criteria were operationalized using Association for Clinical Genomic Science (ACGS) Best Practice Guidelines for Variant Classification in Rare Disease 2024 (v1.2), relevant ClinGen/Sequence Variant Interpretation recommendations, and finalized gene- or disease-specific ClinGen VCEP specifications where available. Evidence strengths were integrated using the Tavtigian Bayesian point system (Supporting = 1 point, Moderate = 2 points, Strong = 4 points, Very Strong = 8 points; Likely Pathogenic = 6–9 points; Pathogenic ≥10 points), with at least two independent evidence lines required for a Pathogenic or Likely Pathogenic classification [28,29,30,31,32,33,34,35,36]. PM2 was applied at Supporting strength [29]. PP5 and BP6 were not used, and Geneyx, ClinVar, gene-specific databases, and literature-search resources were used to identify and trace evidence [28,32]. Explicit case-level or laboratory-series observations within a submission were considered only within the relevant criterion when non-overlap could be assessed; the submission’s aggregate classification was not counted as an independent evidence line. Current-study observations were excluded from external recurrence counts, and relatives contributing segregation evidence were not reused as independent recurrence cases. Variant-specific evidence was curated from exact-allele reports and clinical databases [37,38,39,40,41,42,43,44,45,46], population rarity was assessed in gnomAD v4.1.0 [47], and recurrence, segregation, and variant-specific functional data were reviewed in the relevant original reports [48,49,50,51,52,53,54,55]. Same-residue missense evidence was evaluated separately for PM5 and applied only at Supporting strength when comparator pathogenicity and evidence independence were sufficient. Final categories and retained criteria are summarized in the following tables and Table S2B.

Variant classification and sample-level analytical status were recorded separately. For observations in duplicated *PKD1* sequence, the ACMG/AMP category describes the biological significance of the specified sequence change conditional on authentic *PKD1* attribution, whereas the sample-level status indicates whether the available assay data established that attribution or whether validated locus-resolving confirmation remained necessary [20,26,56].

Study observations were categorized as supported within the assessed scope, confirmation-dependent, externally established comparator/reference findings, or unresolved/non-exclusionary.

SNV/small-indel interpretation was separated from CNV/SV assessment. DRAGEN SV-oriented outputs, FullCoverage_GenePanel reports, and normalized read-depth patterns were inspected at the screening level. Because no assay-matched Panel of Normals or formal CNV validation set was available, panel-derived dosage patterns were not interpreted as standalone diagnostic CNV calls. Clinically relevant dosage findings required MLPA, array-based testing, or another validated dosage method [21,57].

Participants with suspected ADPKD/*PKD1*-related disease or suspected *TSC2*/*PKD1* contiguous-gene deletion underwent MLPA using the SALSA MLPA Probemix P351 *PKD1* assay (MRC Holland, Amsterdam, the Netherlands), according to the manufacturer’s instructions. This assay was used for exon-level *PKD1* dosage assessment and included selected *TSC2* probes relevant to contiguous-gene deletions. Existing SNP-array results were used where available. WES was used selectively for independent short-read reidentification of sequence variants; it was not treated as functional evidence and was not considered fully orthogonal for observations in duplicated *PKD1* sequence. Cases without a conclusive finding were retained as unresolved and directed to mechanism-appropriate follow-up testing.

### 2.8. Statistical Analysis

Analyses were descriptive. Run 2 was the primary dataset for broad interval-, gene-, and base-level characterization, whereas Run 1 provided feasibility context. Results were summarized as counts, proportions, arithmetic means, medians, and ranges. No formal hypothesis testing, diagnostic-yield estimation, or sensitivity/specificity calculation was prespecified.

## 3. Results

### 3.1. Implementation Cohort and Analytical Roles

**Figure 2:**
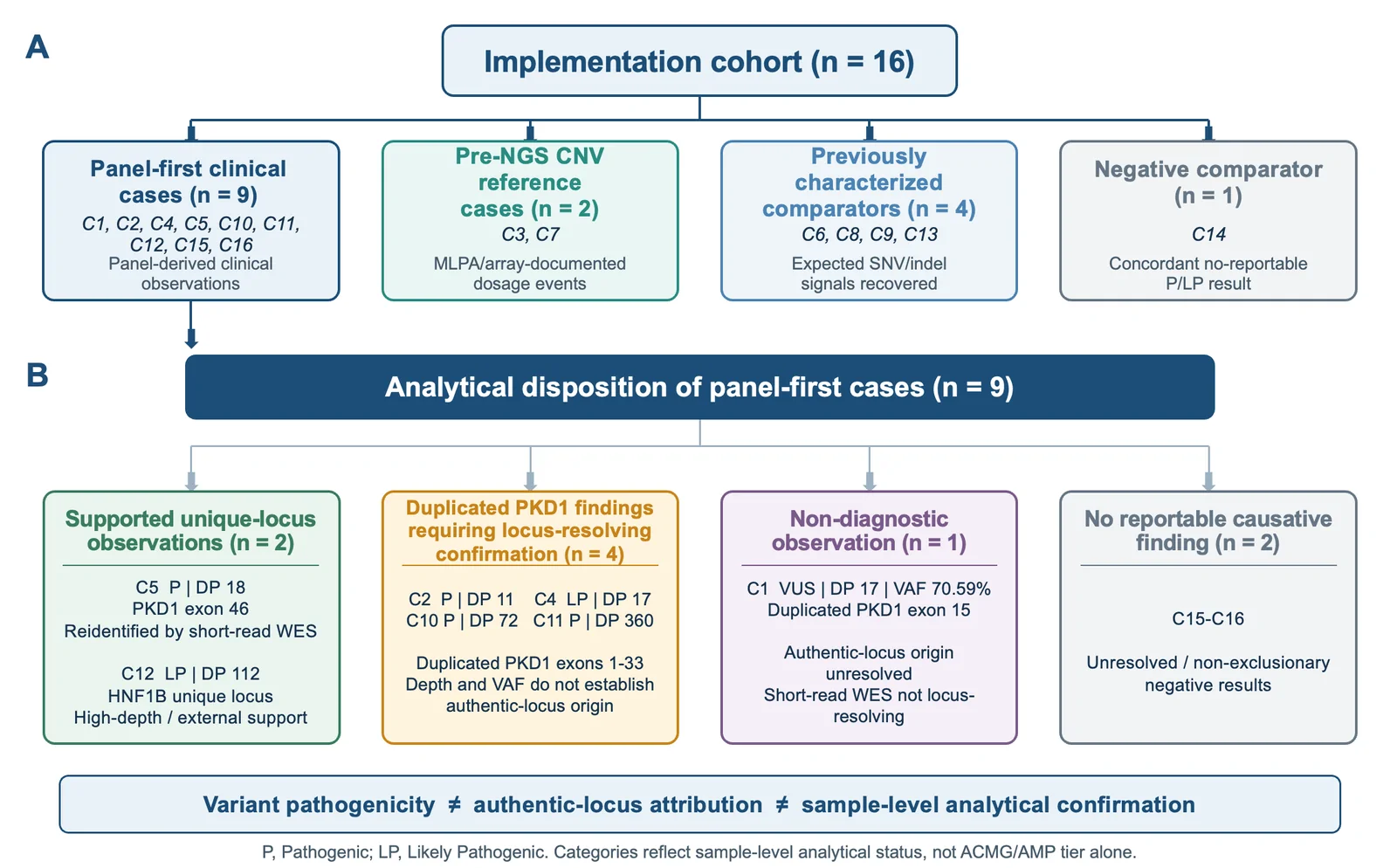
Implementation-set composition and prespecified analytical roles. (**A**) Implementation cohort. (**B**) Analytical disposition of panel-first cases.

The implementation set comprised 16 processed samples assigned to four prespecified analytical roles: nine panel-first clinical cases, two pre-NGS CNV reference cases, four previously characterized sequence-variant comparators, and one negative comparator (Figure 2). These categories distinguished panel-first observations from externally established findings and analytical-boundary cases.

Six panel-first observations involved sequence changes classified as Pathogenic or Likely Pathogenic in *PKD1* or *HNF1B*. Four *PKD1* observations occurred in duplicated sequences and remained confirmation-dependent. C5 was a supported unique-locus *PKD1* observation, independently reidentified by short-read WES, whereas C12 was a supported unique-locus *HNF1B* observation. C1 remained a non-diagnostic VUS, and C15 and C16 remained unresolved/non-exclusionary.

The comparator samples were concordant with their predefined analytical roles. The expected loci were observed in all four sequence-variant comparators, although local panel support varied. C3 and C7 demonstrated different CNV-related analytical boundaries, and C14 had no Pathogenic or Likely Pathogenic observation within the assessed context. Detailed CNV-reference results are presented in Section 3.6.

### 3.2. Run-Specific QC Profiles and Depth Characterization

Run 1 generated a mean of 180,808 reads per sample and served as the initial low-output feasibility configuration. Run 2 generated a mean of 1,176,982 reads per sample and served as the primary analytical dataset. Global mapping remained high in both runs, while the duplicate, multi-mapping and high-MAPQ fractions differed between configurations (Table 2). These descriptors were interpreted together with, but not substituted for, target-specific depth.

### 3.3. Interval- and Gene-Level Depth Completeness

**Figure 3:**
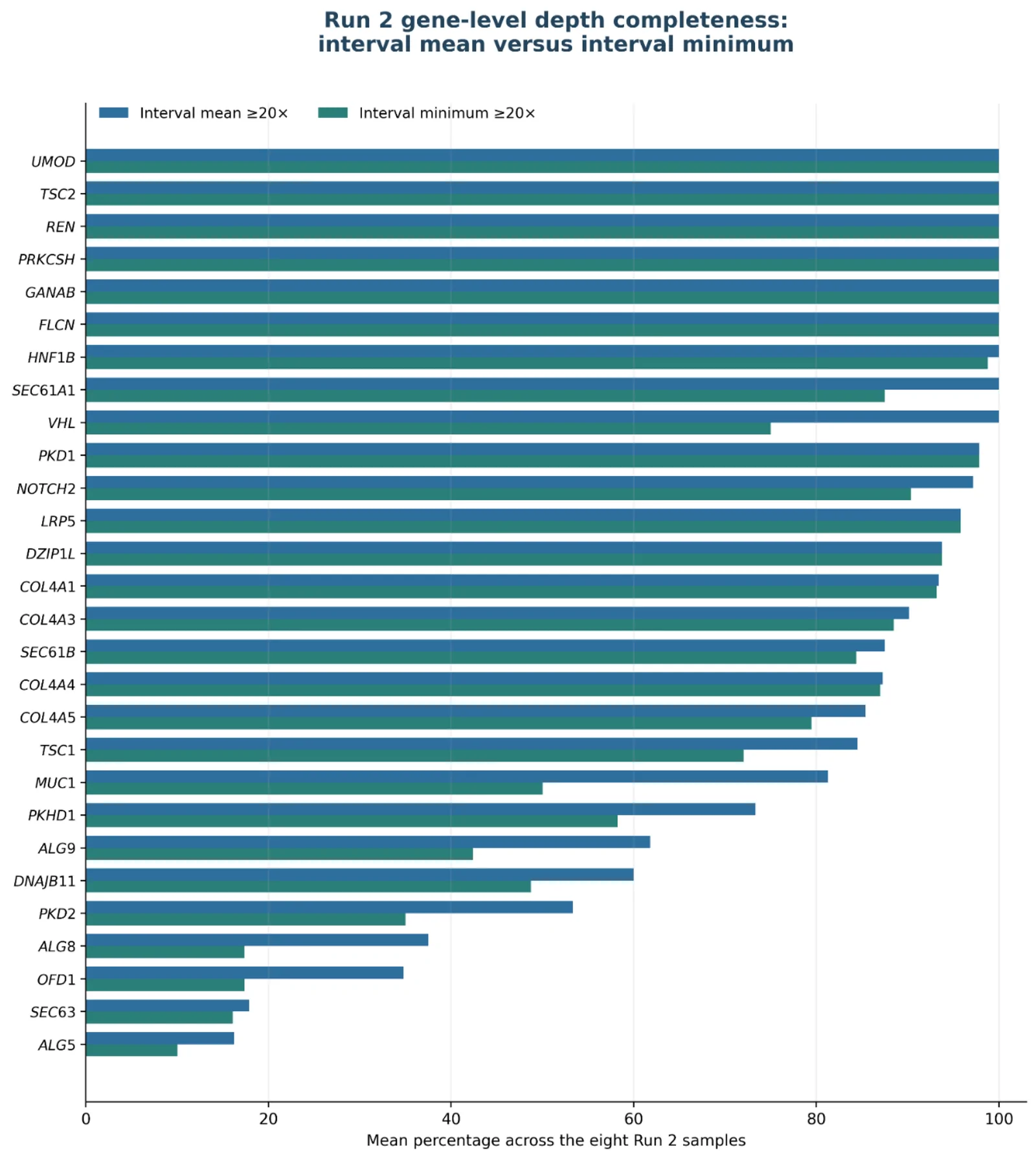
Gene-level aligned-depth completeness in the primary analytical dataset. Bars show the mean percentage of analytical target intervals per gene meeting interval mean depth ≥ 20× and interval minimum depth ≥ 20× across the eight Run 2 samples. These criteria are distinct and do not establish complete gene-wide or authentic-locus callability.

The interval-level framework showed substantial gene-level heterogeneity in aligned-depth completeness (Table 3 and Figure 3). In Run 2, all analytical target intervals in nine genes (*FLCN*, *GANAB*, *HNF1B*, *PRKCSH*, *REN*, *SEC61A1*, *TSC2*, *UMOD*, and *VHL*) had a mean depth of ≥20× in every sample. Under the requirement that the minimum depth of every interval was ≥20×, six genes met the criterion in every sample: *FLCN*, *GANAB*, *PRKCSH*, *REN*, *TSC2*, and *UMOD*. These results document depth completeness within the assessed records and do not establish complete gene-wide callability. Descriptive gene-average aligned depth also varied: the unweighted mean of DRAGEN interval mean coverage values was approximately 270.0× for *PKD1*, 258.8× for *TSC2*, and 209.4× for *PRKCSH*, but 22.8× for *ALG5* and 17.6× for *ALG8*. *PKD1* illustrates why this aggregate is not a completeness endpoint: high gene-average depth coexisted with exon 1 undercoverage and unresolved authentic-locus attribution across duplicated exons 1–33. The complete matrix is provided in Supplementary Table S6A and Supplementary Data File S5. Other genes showed high but incomplete, intermediate, or recurrently weak profiles, defining gene- and interval-specific limits for negative-result interpretation. The *MUC1* summary did not assess its pathogenic coding-VNTR mechanism.

**Figure A2:**
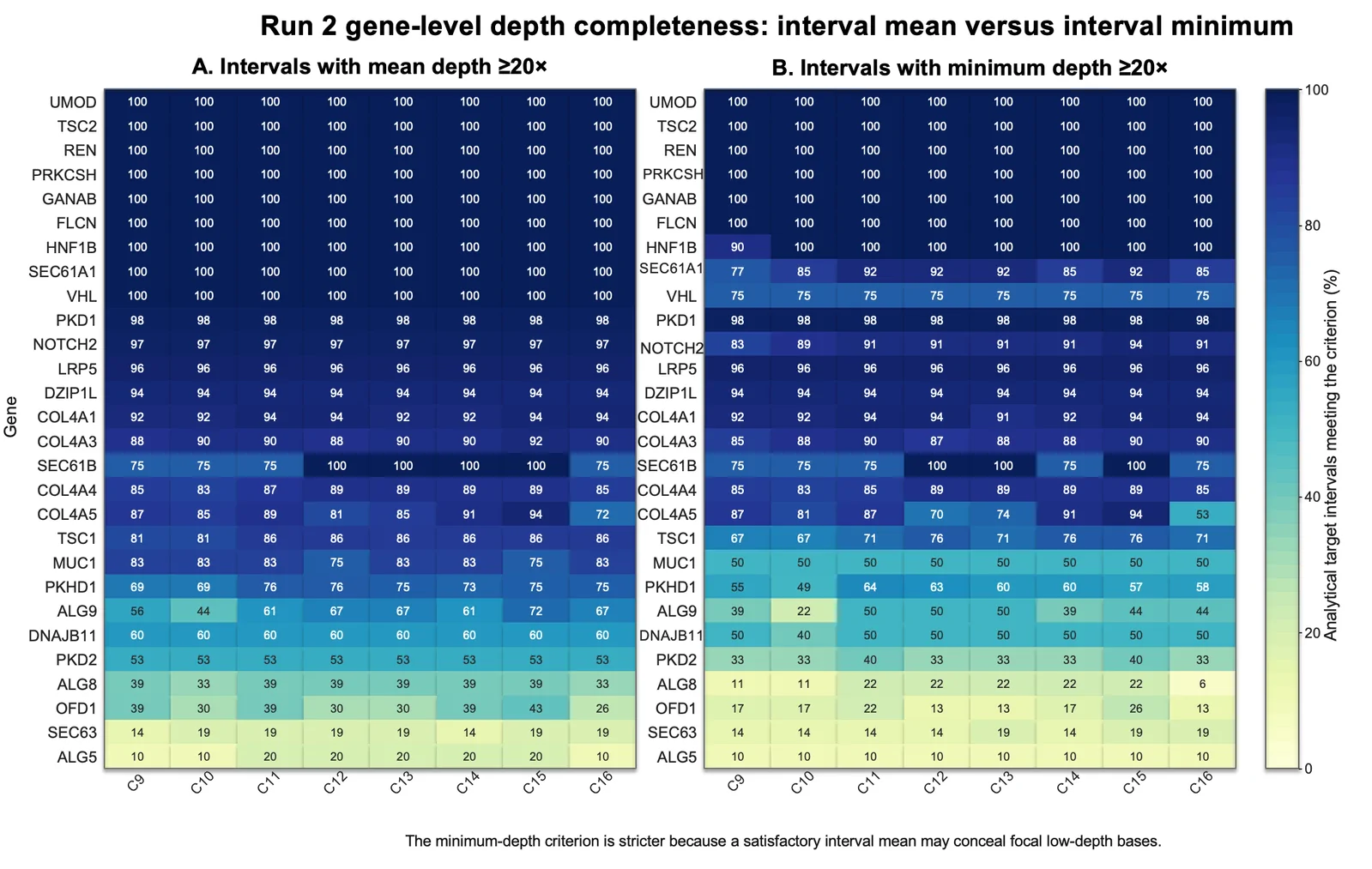
Run 2 sample-by-gene depth completeness using two non-equivalent criteria. Panel (**A**) shows the percentage of analytical target intervals with mean depth ≥20×; Panel (**B**) shows the percentage with minimum depth ≥20×. The minimum-depth criterion is stricter because a satisfactory interval mean can conceal focal low-depth bases.

**Figure A3:**
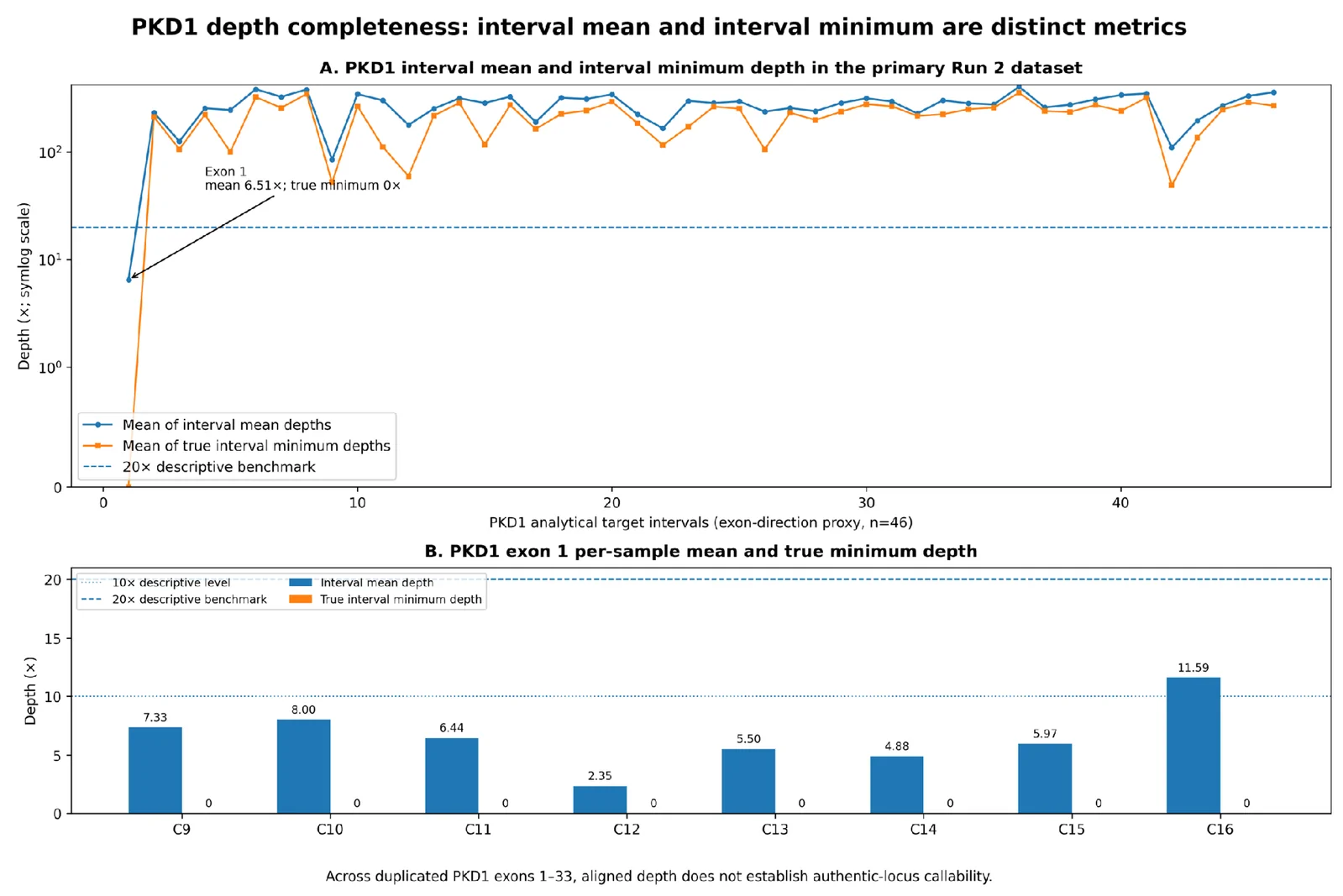
PKD1 depth completeness: interval mean and interval minimum are distinct metrics. (**A**) PKD1 interval mean and interval minimum depths in the primary Run 2 dataset. For each of the 46 PKD1 analytical target intervals, the blue series represents the across-sample mean of the interval mean depths, whereas the orange series represents the corresponding across-sample mean of the true interval minimum depths. The dashed horizontal line indicates the 20× descriptive benchmark. The exon 1 analytical interval had an across-sample mean interval depth of 6.51× and a true interval minimum depth of 0×. (**B**) Per-sample interval mean and true interval minimum depths for the PKD1 exon 1 analytical interval. Blue bars indicate interval mean depth; orange baseline segments indicate a true interval minimum depth of 0× in all samples. The dotted and dashed horizontal lines indicate the 10× descriptive level and the 20× descriptive benchmark, respectively. Across duplicated PKD1 exons 1–33, aligned depth does not establish authentic-locus callability.

*PKD1* showed near-complete aligned-depth completeness in Run 2: 45 of 46 intervals met both the mean-depth and minimum-depth ≥20× criteria in every sample. The exception was the recurrently weak exon 1 interval (chr16:2135474-2135689). Gene-level mean-versus-minimum completeness and *PKD1* interval-level details are shown in Appendix A, Figure A2 and Figure A3; complete matrices are provided in Supplementary Tables S3–S13.

### 3.4. PKD1 Depth Completeness and Limits of Locus Attribution

**Figure 4:**
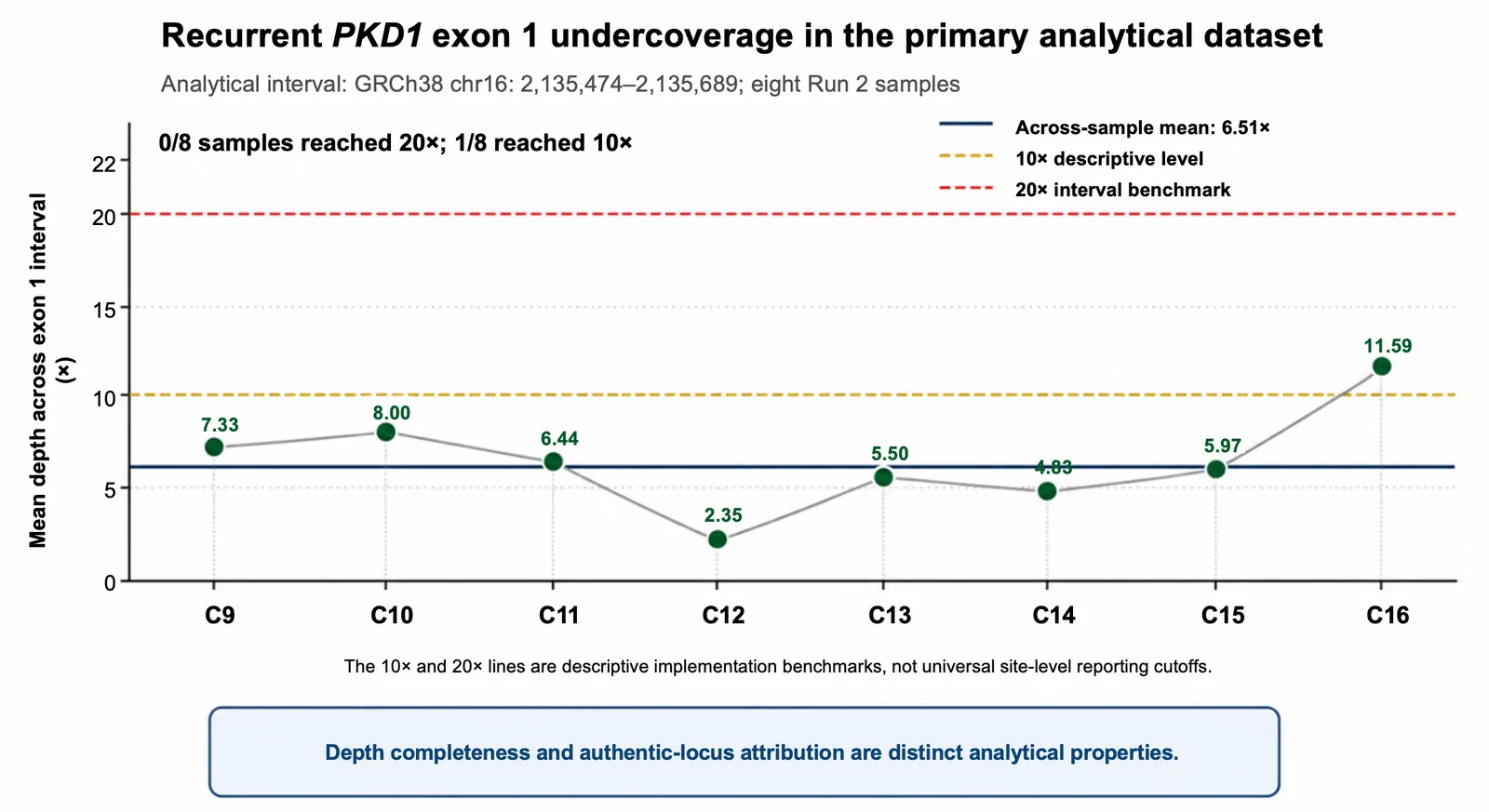
Recurrent *PKD1* exon 1 undercoverage in the primary analytical dataset. Points show sample-specific mean depth for the exon 1 analytical interval. The across-sample mean was 6.51× (range, 2.35–11.59×); 0/8 samples reached a mean interval depth of 20× and 1/8 reached 10×. The true interval minimum was 0× in every sample (Appendix A, Figure A3).

*PKD1* contained 46 analytical target intervals. In all Run 2 samples, 45 intervals met both ≥20× criteria. The recurrent failing interval corresponded to exon 1 (GRCh38 chr16:2135474-2135689), with an across-sample mean interval depth of 6.51× (range, 2.35–11.59×; Figure 4) and a true interval minimum of 0× in every sample (Appendix A, Figure A3). No sample reached a mean depth of 20× at this interval, and only one reached 10×. Exon-proxy intervals 9, 42, 3, 22, and 12 had lower depths than most *PKD1* intervals but met both ≥20× criteria in all eight samples; they were relatively weaker, not failing, intervals.

**Figure 5:**
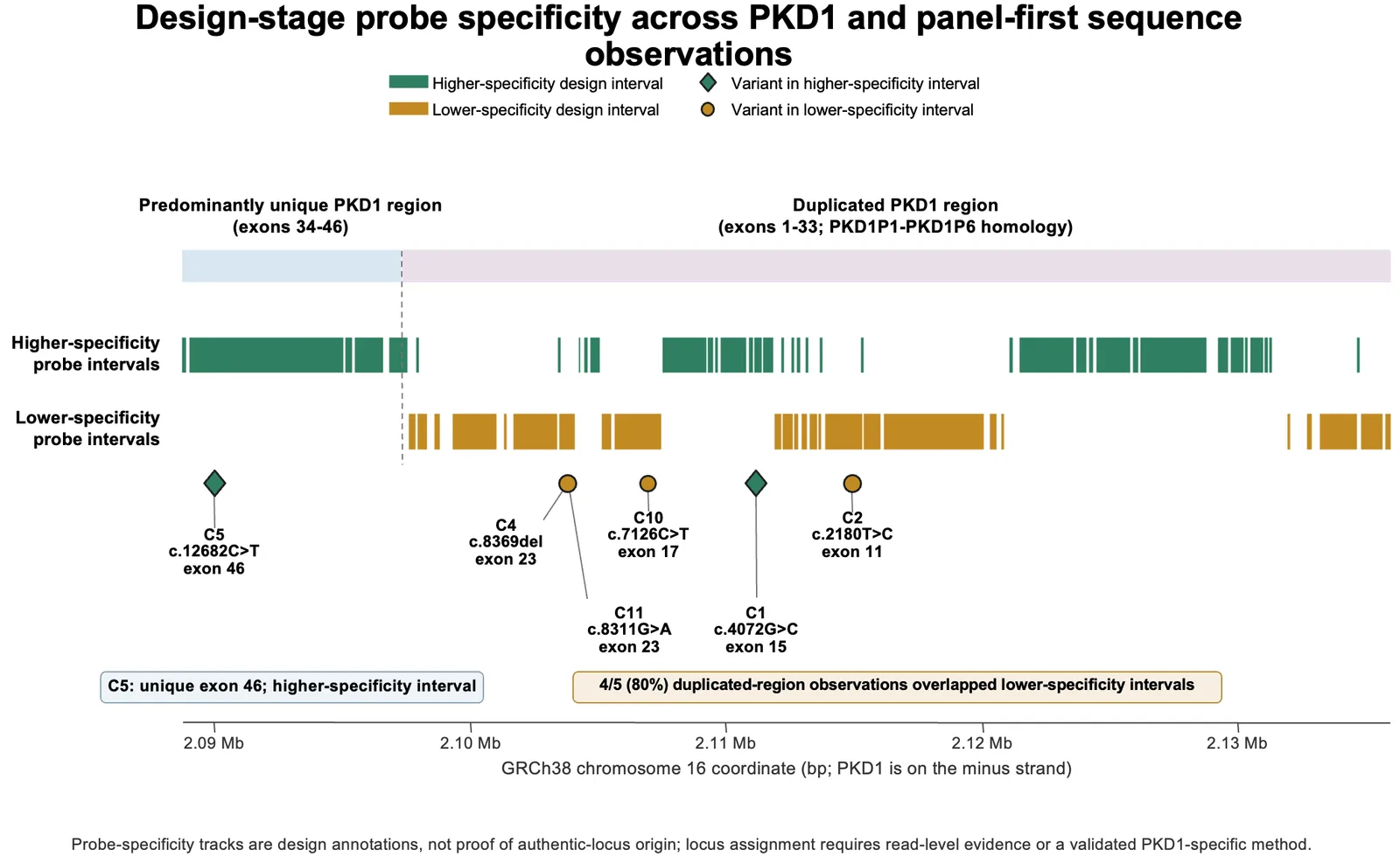
Design-stage probe specificity across *PKD1* and panel-first sequence observations. Variant coordinates were intersected with higher- and lower-specificity design tracks. Four of five duplicated-region observations overlapped lower-specificity intervals. These tracks are design annotations and do not establish authentic-locus origin.

Nominal depth at the remaining *PKD1* intervals did not resolve authentic-locus attribution across duplicated exons 1–33, as summarized in Figure 5. Five panel-first observations were located in this duplicated region: C1, C2, C4, C10, and C11. C2, C4, C10, and C11 overlapped lower-specificity design intervals, whereas C1 overlapped a higher-specificity interval; C5, in unique exon 46, also overlapped a higher-specificity interval. The retrospective competitive FASTQ analysis generated strict locus-restricted REF/ALT fragment counts of 1/3 for C2, 6/6 for C4, 15/8 for C10, and 217/225 for C11, corresponding to limited, moderate, and strong locus support grades. This analysis used a recovery denominator different from nominal DRAGEN DP and was not a validated locus-resolving method. Accordingly, all four Pathogenic or Likely Pathogenic duplicated-region observations remained confirmation-dependent.

### 3.5. Panel-First Sequence Observations: Classification and Analytical Status

Panel-first observations were evaluated using two complementary dimensions: the ACMG/AMP classification of the sequence change and the analytical confidence supporting its detection in the tested sample. Four observations had nominal site-level depth below 20× (C1, C2, C4, and C5). Therefore, the ≥20× interval-mean benchmark was not used as an automatic exclusion threshold for detected candidates. Instead, each site was assessed in context, taking into account depth, allelic balance, read quality, local mapping, variant class, locus context, and independent evidence. Conversely, the absence of a candidate in an interval below this benchmark was considered non-exclusionary.

C5 and C12 represented supported unique-locus observations with diagnostic relevance.

C5 carried *PKD1* c.12682C>T, p.(Arg4228Ter). Although this variant was detected below the descriptive local support benchmark, it was located in a high-specificity region and was independently reidentified in a separate short-read WES dataset, supporting a confident diagnostic interpretation.

C12 carried *HNF1B* c.1091C>G, p.(Ser364Ter), in a gene for which all analytical target intervals met the mean-depth benchmark in the primary run, supporting a clinically coherent *HNF1B*-related diagnosis.

Molecular characteristics, final classifications, retained ACMG/AMP criteria, and sample-level analytical status are shown in Table 4 and Table S2A–D.

### 3.6. CNV Reference Cases and Complementary Evidence

**Figure A4:**
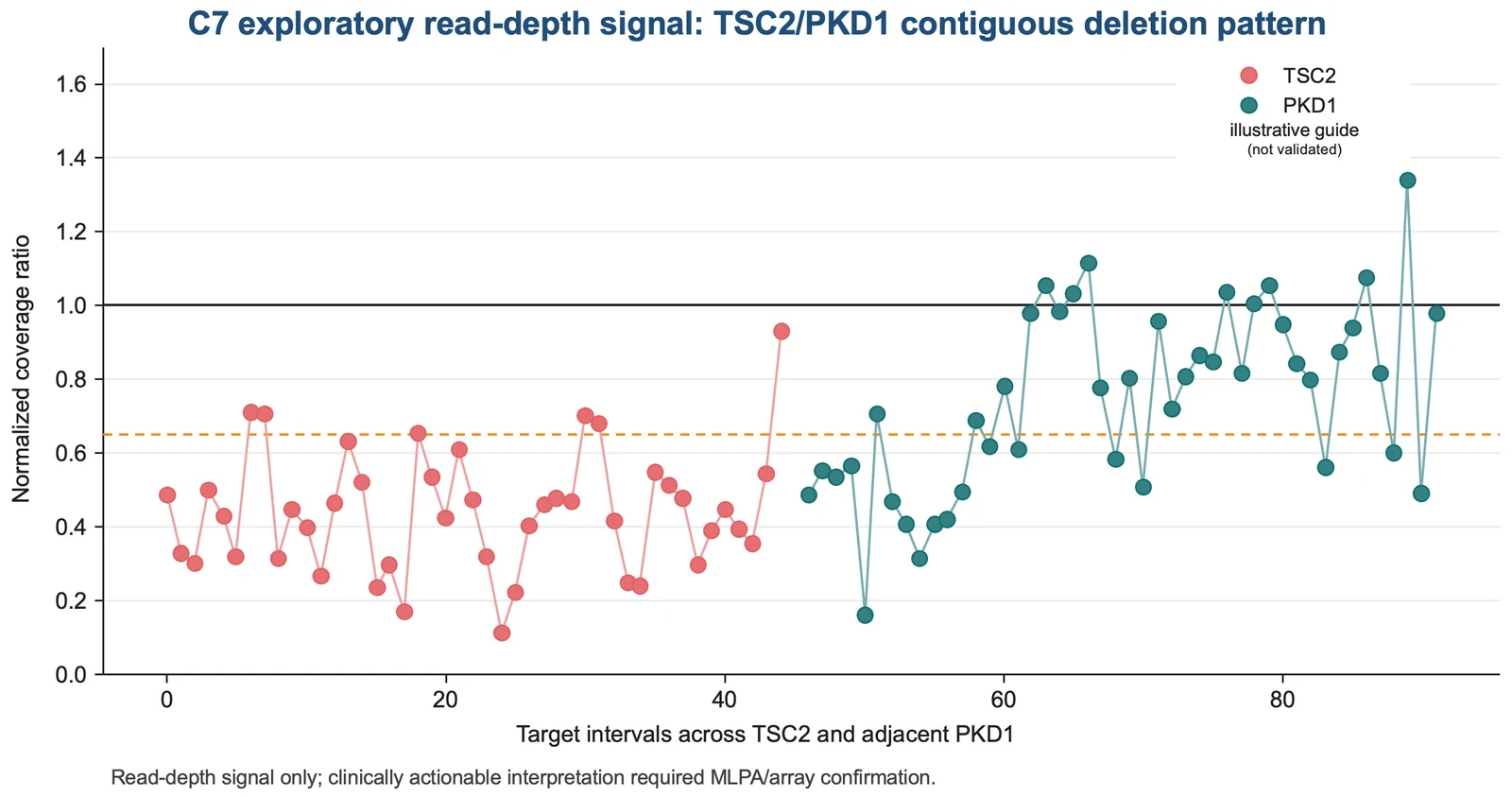
Exploratory read-depth behavior across TSC2 and adjacent PKD1 intervals in C7. The loss-like pattern is concordant with an externally established MLPA/array-confirmed contiguous deletion and is not a standalone validated CNV call or sequence-level breakpoint definition.

**Figure A5:**
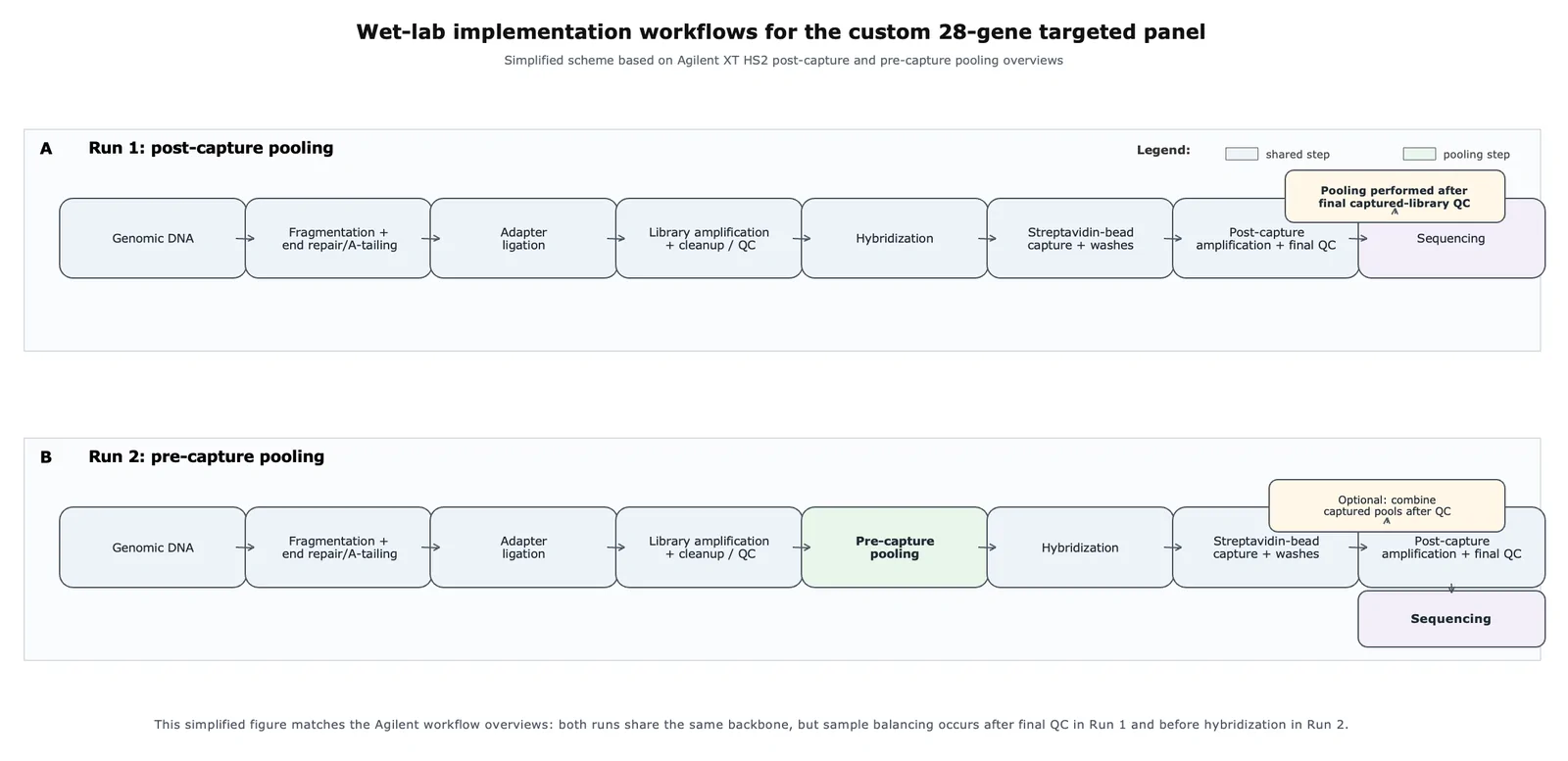
Wet-lab implementation pathways for the focused panel. Both configurations used the Agilent SureSelect XT HS2 workflow and the same custom probe design. (**A**) Run 1 used individual hybridization/capture with post-capture pooling; (**B**) Run 2 used normalized pre-capture pooling before pooled hybridization/capture. Output, pooling stage, and sample composition changed concurrently.

Two pre-NGS MLPA-documented CNV reference cases were included to examine the behavior of known dosage events in panel data. They were not treated as de novo NGS CNV discoveries and did not validate standalone CNV calling. C3 carried a known heterozygous deletion involving *PKD1* exon 1; because the event overlapped the recurrent weak interval, panel read depth did not reliably assess it, and the finding remained supported by MLPA. C7, with an externally established contiguous *TSC2*/*PKD1* deletion, showed a qualitatively concordant loss-like read-depth pattern and was further delineated by array-based copy-number analysis. C7 belonged to the previously reported family [59]. The panel pattern was interpreted as exploratory concordance evidence, not a validated standalone CNV call (Appendix A, Figure A4). In the absence of an assay-matched Panel of Normals and formal CNV validation, clinically relevant dosage findings required MLPA, array analysis, or other validated diagnostic evidence.

## 4. Discussion

### 4.1. From Guideline-Based Testing to an Assay-Specific Evidence Boundary

This pilot study shows why guideline-informed gene selection is necessary but insufficient for clinical implementation. Technical standards require assay-specific characterization of target completeness, homologous-sequence interference, confirmation policy, and residual limitations [17,19]. The fixed set of 694 analytical target intervals made the depth analysis reproducible, but did not convert the pilot into a formal validation study or a universally reportable range.

The analysis distinguished four non-equivalent properties: interval mean depth, interval minimum depth, base-level completeness, and site-level variant support. It also separated aligned depth from authentic-locus attribution in duplicated *PKD1* sequence.

The ≥20× interval-mean threshold was used as a descriptive implementation benchmark, not a universal site-level detection or reporting cutoff. Clinical NGS standards emphasize that thresholds and reportable ranges must be assay-specific and linked to the variant classes and genomic contexts actually validated [17,19]. A detected site below the benchmark could therefore undergo analytical review, whereas a negative finding in the same interval remained non-exclusionary.

### 4.2. Clinical Relevance of a Focused ADPKD-Spectrum Panel

One motivation for developing the dedicated 28-gene panel was to improve the balance among testing scope, laboratory resources, turnaround time, and interpretive workload by using an established short-read hybrid-capture workflow on available MiSeq infrastructure. A focused assay interrogates a smaller genomic footprint, supports multiplexing, concentrates sequencing on clinically selected loci, and limits downstream curation compared with immediate genome-wide testing. KDIGO identifies targeted PKD/nephrology NGS panels as a pragmatic and cost-conscious approach in appropriate ADPKD scenarios, while direct-capture studies show that technically difficult *PKD1* analysis can be integrated into panel workflows when probe design, bioinformatics and complementary confirmation are explicitly curated [1,17,18,19].

This study has practical implications for staged genetic testing workflows. A focused panel may represent an accessible first-line test for selected referrals in the ADPKD spectrum. Cases that remain unresolved, involve low-coverage intervals, include suspected CNVs, or show phenotypes beyond the panel scope should not be interpreted as negative exclusions but should prompt complementary testing.

Genetic-testing studies in CKD and cystic kidney disease demonstrate the utility of targeted approaches to identify expected and overlapping diagnoses, but yield and interpretability are dependent on cohort selection, scope of assay, and classes of variants assessed [12,13,15].

### 4.3. Genes with Strong Aligned-Depth Completeness and Gene-Specific Interpretability

A key result of the primary analytical run was that all defined analytical target intervals in several clinically relevant genes met the ≥20× mean-depth benchmark, and a subset also met the stricter interval-minimum criterion. This identified regions in which aligned-depth evidence was comparatively strong while preserving the distinction between depth completeness and complete gene-wide exclusion. *HNF1B*, *FLCN*, *TSC2*, and *VHL* are particularly relevant to medical practice because their associated disorders may overlap with cystic kidney disease but carry different surveillance, counseling, or dosage-testing implications [6,8,10,11].

Case C12 illustrates this principle. The *HNF1B* nonsense variant c.1091C>G, p.(Ser364Ter), was detected with a VAF of 41.96% and 65/47 REF/ALT reads (DP 112) in a gene for which all analytical target intervals met the mean-depth benchmark in the primary run. In the context of small cystic kidneys, early-onset CKD, young-onset diabetes, and family history, this observation supported a clinically coherent *HNF1B*-related diagnosis. This molecular resolution directly informs patient care and specialized surveillance, such as guiding the management of maturity-onset diabetes of the young (MODY5)—which frequently requires early insulin therapy—and prompting routine screening for highly associated extrarenal manifestations, including hypomagnesemia, hyperuricemia, and pancreatic or hepatic dysfunction [6].

Strong aligned-depth completeness strengthens SNV/indel interpretation within the assessed intervals but does not exclude all disease mechanisms. *HNF1B*-related disease may result from intragenic sequence variants or larger 17q12/*HNF1B* deletions, so adequate sequence depth does not replace dosage assessment when clinically indicated [6].

Similarly, complete reportable-interval coverage of *FLCN*, *VHL* and *TSC2* supports sequence-level interpretation in clinically actionable differential diagnoses with specific surveillance implications, including Birt–Hogg–Dubé syndrome, von Hippel–Lindau disease and tuberous sclerosis complex [8,10,11]. In this assay, depth completeness for *VHL*, *FLCN*, or *TSC2* did not establish the detection of all structural, mosaic, intronic, or regulatory events [17,19,21].

These examples illustrate that negative results are not uniformly informative across a panel. In genes with stronger aligned-depth completeness, negative SNV/indel findings are more informative within the targeted intervals and for the variant classes actually assessed. In contrast, technically constrained loci, incomplete intervals, and mechanisms outside the demonstrated scope require cautious and mechanism-specific interpretation.

### 4.4. Run Profiles and Gene-Specific Depth Boundaries

Run 1 and Run 2 had distinct analytical roles. Run 1 established low-output feasibility, whereas Run 2 provided the primary higher-output dataset. Because sequencing output, pooling stage, and sample composition changed concurrently, the study cannot rank pooling strategies or attribute the depth profile to a single workflow component. The difference between 82.7% of sample-interval observations meeting the interval-mean benchmark and 76.7% meeting the interval-minimum benchmark is analytically important: a satisfactory record mean can conceal focal low-depth bases.

The same custom probe design and SureSelect XT HS2 capture chemistry were retained in both configurations, providing a common descriptive backbone. *PKD1* exon 1 was the clearest persistent pattern: interval mean depth increased from 0–1.27× in Run 1 to 2.35–11.59× in Run 2, but the true interval minimum remained 0× in all 16 samples. The focal gap was therefore not explained by low sequencing output alone and is consistent with an interval-specific limitation related to local sequence context, capture efficiency, or both. This observation does not isolate a causal probe design effect because the design itself was not experimentally varied.

### 4.5. PKD1 Reportability: Depth and Authentic-Locus Attribution Are Distinct

*PKD1* accounts for a large proportion of genetically resolved ADPKD and remains the most technically demanding locus. Published short-read strategies have supported *PKD1* analysis when locus-aware design or locus-specific enrichment, careful alignment, interval-level depth assessment, and complementary *PKD1*-resolving methods are incorporated [16,18,20,26,44].

The probe design strategy involved an explicit trade-off. Restricting the design to higher-specificity intervals would have omitted selected portions of the pseudogene-homologous region. Instead, probe selection uniqueness constraints were relaxed for selected duplicated intervals, and both higher- and lower-specificity probe classes were retained. This preserved sequence representation across exons 1–33 but accepted that lower-specificity probes could enrich both authentic *PKD1* and homologous *PKD1P1–PKD1P6* sequences. Four of the five panel-first observations in duplicated *PKD1* sequences intersected lower-specificity intervals, illustrating the clinically relevant target space that would have been lost under a higher-specificity-only design. These intervals were retained for analysis but were not assumed to be globally callable.

A validated direct-capture strategy showed that this trade-off can be managed within an assay-specific framework. Eisenberger et al. targeted the complete genomic *PKD1* region with 1 kb of flanking sequence, relaxed probe selection settings in duplicated regions from a maximum of three to as many as ten close genomic matches, and increased probe representation 12-fold for exons 1 and 42. Using 250–300-bp mechanically sheared fragments, paired-end 2 × 150-bp sequencing, complete-reference mapping, and conventional confirmation, the workflow was evaluated in 55 previously characterized *PKD1*-positive samples and detected 681/683 verified sequence-variant observations, with reported performance above 98%. Competitive mapping remained problematic at six sites in exons 5, 11, 17, 21, and 26, and candidate or unresolved findings retained locus-specific confirmation [18].

The present pilot adopted the same general principle of retaining lower-specificity probes rather than excluding duplicated sequences, but it was not analytically equivalent to that validated workflow. Capture chemistry, fragmentation, sequencing output, and validation scale differed, and no equivalent positive-control set spanning duplicated *PKD1* was available. The higher- and lower-specificity tracks, therefore, document design intent and expected probe uniqueness, not validated locus-specific sensitivity. Notably, Eisenberger et al. reported that the same exon 11 variant observed in our study, *PKD1 c.2180T>C*, p.(Leu727Pro), was not detected in one sample because it failed the fixed variant-calling filters and was detected in a second only after the ALT-read threshold was lowered from 20% to 8% in a second-step analysis. For the detected occurrence, the authors reported an ALT-read fraction of 10%. In C2, the nominal DRAGEN/Geneyx call had a DP of 11, with 6 REF and 5 ALT reads (VAF 45.45%), showing an approximately balanced nominal allelic representation despite the low depth. This site-specific contrast may reflect workflow-dependent differences in probe design, fragment context, paired-end discrimination, alignment and variant calling, or stochastic sampling at low depth. However, the analytical workflows and read-counting denominators were not equivalent, and only four fragments met the conservative locus-assignment criteria in C2 (3 ALT and 1 REF). Together with the presence of the ALT-equivalent base in at least one homologous pseudogene sequence, these limitations preclude attribution of the difference specifically to individual capture or any claim of superior analytical performance. Duplicated-region observations, therefore, remained subject to locus-aware review and confirmation.

Exon 1 remained a separate depth limitation, consistent with the known difficulty of this GC-rich interval. In a locus-specific LR-PCR/NGS workflow, Kinoshita et al. re-amplified exon 1 from a *PKD1*-specific LR-PCR product and added the corrective-PCR library to the sequencing pool [60]. A similar rescue strategy could be evaluated prospectively for the present assay, but it would require validation before clinical use. Targeted long-read approaches provide another option for unresolved pseudogene-homologous, deep intronic, indel, and copy-number findings [16].

Therefore, negative targeted short-read panel results should be interpreted according to the scope of the evaluation and not as a blanket exclusion. Mechanism-directed complementary testing may be required for constrained *PKD1* intervals, pseudogene-related events, deep intronic or complex structural variants, and low-level mosaicism [1,16,21]. Residual dosage questions require validated CNV testing [57], whereas suspected ADTKD-*MUC1* requires dedicated *MUC1* analysis [61].

### 4.6. Beyond PKD1: Other Gene- and Mechanism-Specific Boundaries

The principal additional aligned-depth limitations were not confined to *PKD1*. In Run 2, *ALG5*, *SEC63*, *OFD1*, and *ALG8* remained recurrently weak, with mean-depth/minimum-depth completeness of 16.2%/10.0%, 17.9%/16.1%, 34.8%/17.4%, and 37.5%/17.4%, respectively. Negative results in these genes are therefore non-exclusionary within this pilot. *PKD2*, *DNAJB11*, *ALG9*, and *PKHD1* showed intermediate completeness, while several other genes had high but incomplete profiles. These differences should be communicated at the gene and interval level rather than hidden by panel-wide averages.

A second boundary concerned molecular mechanisms not captured by ordinary exon-focused short-read depth. The *MUC1* coding-VNTR was outside the demonstrated scope and requires dedicated testing when ADTKD-*MUC1* is suspected [61]. Likewise, interval depth does not establish detection of every deep intronic, repeat-mediated, structural, splice-altering, or mosaic event [1,17,19,21]. These limitations are independent of whether a gene appears nominally present in the design.

A third boundary concerned dosage analysis. The C7 reference case showed that read-depth patterns can provide concordance evidence, but the absence of an assay-matched Panel of Normals and formal CNV validation prevented standalone diagnostic CNV calling. Consequently, CNV interpretation remained mechanism-directed and dependent on MLPA, array analysis, or another validated dosage method [21,57].

### 4.7. Variant Classification, Sample-Level Confidence, and Complementary Testing

ACMG/AMP classification describes the pathogenic significance of a specified sequence change; it does not by itself establish that the tested sample carries that change at the stated locus. Conversely, adequate analytical support for an observation does not determine the biological classification of the sequence change. Reporting these two conclusions separately is particularly important for duplicated *PKD1* sequences [20,26,35,56].

The panel is therefore best positioned as the first component of a staged diagnostic workflow. Within a formally validated implementation, adequately supported unique-locus SNV/indel observations could be handled under the laboratory’s reporting policy. Observations in duplicated-region *PKD1* require a validated locus-resolving approach, such as *PKD1*-specific long-range PCR followed by Sanger or NGS, a prospectively validated locus-aware assay, or targeted long-read sequencing.

Dosage questions require particular caution. Read-depth patterns from targeted NGS may provide useful screening or concordance evidence, but they should not be treated as standalone diagnostic CNV calls unless CNV performance has been specifically validated for the assay. Constitutional CNVs should be interpreted according to ACMG/ClinGen technical standards; clinically relevant panel-derived dosage findings should be confirmed with a validated orthogonal method when the panel’s CNV performance has not been established [21,57]. MLPA or another validated CNV assay may be appropriate, and array-based analysis may be suitable for larger events.

If clinical suspicion persists in the face of a negative or non-diagnostic result, mechanism-directed follow-up testing should be performed. Depending on the residual question, this may include broader WES or WGS, validated *PKD1*-specific or targeted long-read sequencing, mosaicism-aware testing if depth allows, or family-based studies [1,16,21]. Residual dosage questions require validated CNV testing [57], whereas suspected ADTKD-*MUC1* requires dedicated *MUC1* analysis [61].

### 4.8. Limitations and Priorities for Formal Validation

The study has several limitations. The cohort was small and deliberately heterogeneous, and it was not designed to estimate diagnostic yield. The two runs had distinct sample compositions and sequencing configurations; enrichment metrics such as panel-specific on-target rate and Fold-80 base penalty were unavailable; and the retrospective locus-aware FASTQ analysis and CNV analysis lacked a validated assay-matched reference set.

Formal validation should include larger positive and negative control sets, repeatability and reproducibility studies, reference materials, variant-class-specific sensitivity and precision, and an assay-matched CNV reference model [19,20,21]. Targeted optimization or a validated rescue strategy should be evaluated separately for *PKD1* exon 1 [60]. Until those steps are completed, the panel should remain a transparently bounded first component of a staged diagnostic pathway.

Despite these limitations, the data provide a practical roadmap for subsequent validation and optimization. Key priorities are local rescue of *PKD1* exon 1, expanded locus-resolving positive controls across duplicated *PKD1* sequence, repeatability and reproducibility studies, and prospective validation of SNV/indel and CNV performance under fixed laboratory and bioinformatic conditions [19,21,62]. The fixed 694-record denominator can support this future work, but it is not itself a validated clinical reportable range.

## 5. Conclusions

This pilot implementation study defined an assay-specific analytical scope for a focused 28-gene ADPKD-spectrum panel using a fixed denominator of 694 analytical target-interval records. In the primary dataset, nine genes had all interval means at ≥20× and six also met the stricter interval-minimum criterion, while depth completeness remained gene- and interval-dependent. *PKD1* showed two distinct limitations: persistent exon 1 undercoverage and unresolved authentic-locus attribution across duplicated sequences. Four Pathogenic or Likely Pathogenic *PKD1* observations, therefore, remained confirmation-dependent. These findings support a staged implementation model built on three linked requirements: first, nominal gene content must be converted into an empirically demonstrated reportable range; second, residual weak intervals must be explicitly disclosed; and third, orthogonal or complementary testing must be used whenever the remaining diagnostic question falls outside the assay’s demonstrated analytical range.

## Figures and Tables

**Table 1:** Case categories and their role in the pilot implementation.

Category	Cases	Role in the Study
Panel-first clinical cases	C1, C2, C4, C5, C10, C11, C12, C15, C16	Real-world implementation and case-level interpretation
Pre-NGS MLPA-documented CNV reference cases	C3, C7	Known dosage events
Previously characterized positive comparators	C6, C8, C9, C13	Comparator findings used for concordance and boundary interpretation
Previously characterized negative comparator	C14	Known negative comparator

**Table 2:** Run-specific laboratory configurations, global QC profiles, and depth metrics.

Metric	Run 1	Run 2
Analytical role	Initial low-output feasibility configuration	Primary higher-output analytical configuration
Samples	C1–C8; *n* = 8	C9–C16; *n* = 8
MiSeq configuration	Nano v2; paired-end 2 × 150 bp	Micro v2; paired-end 2 × 150 bp
Pooling stage	Individual hybridization/capture; post-capture pooling	Normalized pre-capture pooling; pooled hybridization/capture
Reads/sample, mean (range)	180,808 (127,534–261,164)	1,176,982 (859,420–1,484,722)
Q30 bases, mean	88.77%	92.35%
Mapped reads, mean	98.59%	98.04%
Properly paired reads, mean	97.98%	97.72%
Duplicate reads, mean	1.06%	4.64%
Multi-mapping reads, mean	10.13%	29.13%
Reads with MAPQ ≥ 40, mean	83.80%	60.45%
Length-weighted mean interval depth	11.6×	145.5×
Interval records with mean depth ≥ 20×, pooled count/proportion	463/5552 (8.3%)	4592/5552 (82.7%)
Interval records with minimum depth ≥ 20×, pooled count/proportion	158/5552 (2.8%)	4256/5552 (76.7%)
Interval record bases covered at ≥10×, pooled count/proportion	437,190/824,600 (53.0%)	739,087/824,600 (89.6%)
Intervals below 20× mean depth/sample	636.1/694	120.0/694
Intervals below 20× minimum depth/sample	674.2/694	162.0/694

**Table 3:** Selected gene-level aligned-depth completeness in the primary Run 2 dataset.

Gene	Analytical Target Intervals (*n*)	Mean ≥20×	Minimum ≥20×	Bases ≥10×	Interpretation
*PKD1*	46	97.8%	97.8%	98.9%	45/46 intervals met both criteria; exon 1 remained a recurrent limitation; duplicated exons were not declared globally callable.
*PKD2*	15	53.3%	35.0%	68.3%	Intermediate completeness; negative findings remain non-exclusionary in incomplete intervals.
*DNAJB11*	10	60.0%	48.8%	61.5%	Intermediate completeness; negative findings remain non-exclusionary in incomplete intervals.
*GANAB*	25	100.0%	100.0%	100.0%	All defined analytical intervals met both depth criteria.
*HNF1B*	10	100.0%	98.8%	100.0%	All interval means met the benchmark; one interval in one sample had minimum depth <20×.
*TSC2*	45	100.0%	100.0%	100.0%	All defined analytical intervals met both depth criteria.
*COL4A3*	52	90.1%	88.5%	95.6%	High but incomplete completeness.
*COL4A4*	47	87.2%	87.0%	94.7%	High but incomplete completeness.
*COL4A5*	53	85.4%	79.5%	98.3%	High but incomplete completeness.
*MUC1*	12	81.2%	50.0%	78.6%	Depth does not assess the coding-VNTR disease mechanism.
*FLCN*	13	100.0%	100.0%	100.0%	All defined analytical intervals met both depth criteria.
*ALG5*	10	16.2%	10.0%	25.6%	Recurrently weak; a negative result is non-exclusionary.
*SEC63*	21	17.9%	16.1%	28.5%	Recurrently weak; a negative result is non-exclusionary.
*OFD1*	23	34.8%	17.4%	51.0%	Recurrently weak; a negative result is non-exclusionary.

Note: Mean ≥ 20× and minimum ≥ 20× are across-sample proportions of gene-assigned analytical target records. The minimum-depth criterion is stricter because it requires every base within an interval record to remain at or above 20×. These descriptors do not establish complete gene-wide or authentic-locus callability.

**Table 4:** Molecular characteristics, final ACMG/AMP classification, and sample-level analytical status of panel-first pathogenic or likely pathogenic observations.

Case and Sequence Change	Local Short-Read Support	Final ACMG/AMP Class	Evidence Used	Sample-Level Analytical Status
C2—*PKD1* NM_001009944.3:c.2180T>C p.(Leu727Pro)	DP 11; REF/ALT 6/5; VAF 45.45%	Pathogenic 11 points	PS4_VeryStrong + PP1_Supporting + PM2_Supporting + PM5_Supporting	Confirmation-dependent. Duplicated/lower-specificity *PKD1* sequence; validated locus-resolving confirmation is required.
C4—*PKD1* NM_001009944.3:c.8369del p.(Pro2790ArgfsTer85)	DP 17; REF/ALT 10/7; VAF 41.18%	Likely Pathogenic 9 points	PVS1_VeryStrong + PM2_Supporting	Confirmation-dependent. Duplicated *PKD1* sequence and a local G-rich repeat require validated locus-resolving confirmation.
C5—*PKD1* NM_001009944.3:c.12682C>T p.(Arg4228Ter)	DP 18; REF/ALT 11/7; VAF 38.89%	Pathogenic 10 points	PVS1_Strong + PS4_Moderate + PP1_Moderate + PS3_Supporting + PM2_Supporting	Supported unique-locus observation. Independently reidentified by short-read WES; reproducibility support, not functional evidence.
C10-P9—*PKD1* NM_001009944.3:c.7126C>T p.(Gln2376Ter)	DP 72; REF/ALT 47/25; VAF 34.72%	Pathogenic 11 points	PVS1_VeryStrong + PS4_Moderate + PM2_Supporting	Confirmation-dependent. Nominal DP/VAF do not establish authentic *PKD1* attribution in duplicated sequence.
C11-R13—*PKD1* NM_001009944.3:c.8311G>A p.(Glu2771Lys)	DP 360; REF/ALT 175/185; VAF 51.39%	Likely Pathogenic 6 points	PS4_Moderate + PP1_Moderate + PS3_Supporting + PM2_Supporting	Confirmation-dependent. High local support does not replace validated locus-resolving confirmation.
C12-WES7—*HNF1B* NM_000458.4:c.1091C>G p.(Ser364Ter)	DP 112; REF/ALT 65/47; VAF 41.96%	Likely Pathogenic 9 points	PVS1_VeryStrong + PM2_Supporting	Supported unique-locus observation with a clinically coherent *HNF1B*-related phenotype.

Note. Evidence strengths and points follow the ACMG/AMP 2015 framework, ACGS 2024 rare-disease operational guidance, relevant ClinGen/SVI criterion-specific recommendations, and the Tavtigian point system [28,29,30,31,32,33,34,35,36]. Aggregate database classifications and software-generated suggestions were not counted as independent evidence. For duplicated-region *PKD1* observations, molecular classification was reported separately from sample-level authentic-locus confidence. Sample-level details are provided in Supplementary Table S2A–D. For C2, p.(Leu727Arg) provided the same-residue comparator; PM5 was conservatively capped at supporting strength [40,58].

## Data Availability

Aggregated and deidentified analytical data are provided in the manuscript and Supplementary Materials. The design footprint, probe specificity tracks, 694-record analytical BED, de-identified coverage matrices, and run-level summaries are provided. Supplementary Code S1 (v1.0.0) and its documentation are also provided. Germline FASTQ/BAM files are not publicly deposited because they may permit participant re-identification; access may be considered under institutional approval, data protection requirements, and an appropriate data-use agreement.

## Supplementary Materials

The following supporting information can be downloaded at https://www.mdpi.com/article/10.3390/diagnostics16172770/s1, The supplementary package includes Supplementary Methods; Table S1–S13; Supplementary Data Files S1–S5; Supplementary Protocol S1; and Supplementary Code S1 (v1.0.0). References [63,64,65] are cited in the supplementary materials.

## Appendix A

Figure A1, Figure A2, Figure A3, Figure A4 and Figure A5 provide design, depth completeness, CNV concordance, and wet-lab workflow details cited in the main text.
