# Supplementary material for: From Guidelines to Practice: Pilot Implementation and Analytical Boundaries of a Focused ADPKD-Spectrum Gene Panel
Source: Diagnostics (Basel). 2026 Aug 28;16(17):2770. doi: 10.3390/diagnostics16172770 (PMC13564536; doi:10.3390/diagnostics16172770)
Supplement: Supplementary file 1 [file diagnostics-16-02770-s001.zip › diagnostics-4452638_supplementary.pdf]

Supplementary Methods

The supplementary methods describe the gene-specific target architecture and design files, fixed analytical denominator and coverage definitions, laboratory configurations, bioinformatic processing and annotation, coverage metrics, variant classification, and CNV/SV review. Table S1, Tables S2A–S2D, and Tables S3–S13 provide the gene-specific design request, sample-level and molecular findings, locus-aware reassessment, depth-completeness matrices, and global quality-control descriptors. Figures A1–A5 are presented in Appendix A of the main manuscript.

Gene-specific target architecture and design files

Target architecture was specified in Agilent SureDesign against GRCh38. The Entire Transcribed Region option was requested for *PKD1*, *PKD2*, *TSC1*, and *TSC2*; within the selected transcript models this encompassed coding and non-coding exons, introns, and 5′/3′ untranslated regions. *DNAJB11*, *HNF1B*, and *UMOD* were requested as exons plus untranslated regions and 10-bp intronic flanks; most other genes were requested as coding exons plus 10-bp intronic flanks. Additional flanking and intronic probes were included for *HNF1B*. Within pseudogene-homologous *PKD1* sequence, probe-selection uniqueness constraints were relaxed for selected intervals to preserve target representation. Higher- and lower-specificity intervals were retained as separate design-stage tracks; neither class was treated as evidence of authentic-locus origin.

Supplementary Data File S1 provides the machine-readable 28-gene target-region BED; Supplementary Data Files S2 and S3 provide the higher- and lower-specificity design tracks. Supplementary Data File S4 provides the harmonized 694-record analytical target BED, Supplementary Data File S5 provides the complete 694-record × 16-sample depth workbook, and Appendix A, Figure A1 illustrates the design-stage *PKD1* tracks.

Analytical denominator and coverage definitions

Coverage metrics were calculated from 16 DRAGEN FullCoverage_GenePanel exports generated during the study. Each export contained the same 705 ordered records; 11 *PTEN* records outside the final 28-gene scope were excluded, leaving 694 analytical target-interval records per sample. All retained records were included in every calculation. Their row-wise summed length was 103,075 bp and their non-redundant merged union was 102,042 bp. Each run therefore comprised 8 × 694 = 5,552 sample–interval observations and 824,600 interval-record bases. Interval mean depth is the arithmetic mean of base depths within a DRAGEN record; interval minimum depth is the lowest depth anywhere in that record. Length-weighted mean interval depth was calculated as Σ(mean depth × interval length)/Σ(interval length). Per-sample outputs included the proportions of records with mean depth ≥10×, ≥20×, and ≥30×; minimum depth ≥10× and ≥20×; and interval-record bases at ≥10×, calculated as 1 − Σ(bases below 10×)/Σ(interval length). Gene-average aligned depth was calculated separately as the unweighted arithmetic mean of interval Mean Coverage values assigned to a gene. Site-level DP, REF/ALT counts, and VAF were assessed separately. The ≥20× criteria were descriptive implementation benchmarks rather than validated reporting thresholds.

Wet-lab implementation

Genomic DNA was extracted from EDTA-anticoagulated peripheral blood using the Wizard Genomic DNA Purification Kit (Promega), quantified using a Qubit dsDNA High Sensitivity assay, and used at 10–200 ng per library. Both runs used Agilent SureSelect XT HS2 DNA chemistry and the same custom biotinylated RNA-probe design. Enzymatic fragmentation was performed at 37 °C for 10 min followed by 65 °C for 5 min, targeting approximately 180–250-bp genomic inserts for paired-end 2 × 150-bp sequencing; measured post-amplification library-molecule distributions were approximately 300–450 bp. The workflow comprised end repair/A-tailing, adaptor ligation, AMPure XP purification, indexed pre-capture amplification, hybridization, streptavidin-bead capture and washing, post-capture amplification, final purification, and library QC. Run 1 used individual hybridization/capture and post-capture pooling before MiSeq Nano v2 sequencing. Run 2 used normalization and pooling before pooled hybridization/capture and MiSeq Micro v2 sequencing. Supplementary Protocol S1 provides reagent identifiers, reaction volumes, thermal programs, QC points, and the RNase-free transition at hybridization.

Bioinformatic processing and annotation

Paired FASTQ files were processed with Illumina DRAGEN v4.4.4 against GRCh38. Tertiary analysis was performed in Geneyx Analysis using annotation Version Set v6.2. The annotation environment included 1kGenome 2019-02, ACMG 2025-06, ACMG-SV 2025-06, AlphaMissense v2-t114, CADD v1.7, CamouflagedGenes v1, ClinGen 2024-12, ClinVar 2025-06-08, dbNSFP 4.4a, dbSNP b154, gnomAD exomes 2.1.1, gnomAD genomes 3.1.2, gnomAD v4 exomes 4.1.0, gnomAD v4 genomes 4.1.0, MANE v1.4, MasterMind 2025.04.19, OMIM 2025-06, REVEL 2016-03, SnpEff v5.2b.gx3-2025-06, and SpliceAI 1.3. Candidate observations were manually reviewed in Genome Browser and IGV. Software suggestions were treated as decision support and were not accepted without source-level review.

Site-specific locus-aware reassessment of duplicated *PKD1 observations*

The paired FASTQ files for C2, C4, C10, and C11 were retrospectively reassessed with a custom Python 3.12.13 workflow. Exact allele-spanning 11-mer seeds were used only to retrieve candidate pairs. Each pair was scored against matched REF-state and ALT-state haplotypes for authentic *PKD1* and *PKD1P1–PKD1P6*. Exact edit distances used edlib v1.3.9.post1 and competitive mapping summaries used mappy/minimap2 v2.30. A fragment contributed to the conservative locus-restricted count only when the candidate base or indel flanks had Phred quality ≥30, non-candidate paralogous sequence variants excluded all compatible pseudogene competitors represented at the site, authentic *PKD1* was the unique best-scoring locus with an edit-distance margin ≥2, and the read pair showed coherent inward-facing geometry. Counts used a different recovery denominator from nominal DRAGEN DP. The analysis was retrospective and site-specific and did not replace validated locus-resolving confirmation. These counts were interpreted in the context of published *PKD1* short-read sequencing limitations and were not treated as a substitute for validated locus-resolving confirmation [18,20,60].

Variant classification, CNV/SV review, and complementary testing

ACMG/AMP 2015 was used as the overarching five-tier framework. Criteria were operationalized using ACGS 2024 rare-disease guidance, relevant ClinGen/SVI criterion-specific recommendations, and finalized gene- or disease-specific ClinGen VCEP specifications where available. Evidence strengths were integrated using the Tavtigian Bayesian point system [28–31,33–36].

Supporting, Moderate, Strong, and Very Strong pathogenic evidence were assigned 1, 2, 4, and 8 points, respectively; Likely Pathogenic required 6–9 points and Pathogenic ≥10 points, with at least two independent evidence lines. PM2 was retained at Supporting strength; PP5 and BP6 were not used. ClinVar, Geneyx, gene-specific databases, and literature-search resources were used to identify and trace evidence, not as independent criteria. Explicit case-level or laboratory-series observations within a submission were considered only within the relevant criterion when non-overlap could be assessed; the submission's aggregate classification was not counted as an independent evidence line. Current-study observations were excluded from external recurrence counts, and relatives contributing segregation evidence were not reused as independent recurrence cases.

Final categories, retained criteria, and principal variant-specific sources are reported in Table S2B; Table S2C summarizes case-level interpretability, and Table S2D reports the site-specific locus-aware FASTQ reassessment. Biological classification and sample-level analytical status were evaluated separately.

Mechanism-specific limitations

The standard short-read capture workflow did not assess the pathogenic *MUC1* coding-VNTR mechanism; suspected ADTKD-*MUC1* therefore requires dedicated testing [61]. Depth summaries also do not establish detection of every deep intronic, repeat-mediated, structural, splice-altering, or low-level mosaic event. Negative findings were interpreted only within the demonstrated interval-, locus-, variant-class-, and mechanism-specific scope.

Tables S1-S13

Table S1. Gene-specific regions requested in the custom 28-gene capture design

**Gene(s)**	**Requested target category**	**Interpretive note**
PKD1, PKD2, TSC1, TSC2	Entire Transcribed Region	Selected-transcript genomic span including coding/non-coding exons, introns, and 5′/3′ UTRs. The broad request does not imply validated reportability of every included base.
DNAJB11, HNF1B, UMOD	Exons + UTRs + 10-bp intronic flanks	Additional flanking/intronic probes were included for *HNF1B* to support contextual exon-level depth review.
GANAB, PRKCSH, ALG5, ALG8, ALG9, PKHD1, DZIP1L, COL4A1, REN, SEC61A1, SEC63, LRP5, SEC61B, VHL, COL4A3, COL4A4, COL4A5, MUC1, FLCN, NOTCH2, OFD1	Coding exons + 10-bp intronic flanks	Default design request for the remaining genes. *The MUC1 coding VNTR was not assessed by this standard short-read workflow.*

Table S2A. Clinical and molecular characteristics of the implementation samples

Case	Sex / age	Clinical indication / phenotype	Gene	Transcript	Nucleotide change / CNV	Protein change	Variant type / zygosity
C1	M / 31	Typical ADPKD with preserved renal function, increased TKV and positive family history; molecular clarification relevant for prognosis/reproductive counseling.	*PKD1*	NM_001009944.3	c.4072G>C	p.(Ala1358Pro)	Missense / Heterozygous
C2	F / 42	Familial hepatorenal cystic disease with atypical ADPKD/*TSC2-PKD1 differential diagnosis.*	*PKD1*	NM_001009944.3	c.2180T>C	p.(Leu727Pro)	Missense / Heterozygous
C3	F / 27	Familial ADPKD; bilateral renal cysts with very large kidneys, CKD G3.	*PKD1*	*PKD1* / dosage locus	Heterozygous deletion involving the *PKD1 5′* UTR/exon 1 (MLPA-defined; exact breakpoints not established)	Not applicable	CNV deletion / Heterozygous
C4	M / 38	Early-onset rapidly progressive ADPKD with very large kidneys, CKD G4 and negative family history.	*PKD1*	NM_001009944.3	c.8369del	p.(Pro2790ArgfsTer85)	Frameshift deletion / Heterozygous
C5	F / 24	Young-onset ADPKD with preserved renal function, hepatic cysts and strong family history including aneurysmal death.	*PKD1*	NM_001009944.3	c.12682C>T	p.(Arg4228Ter)	Nonsense / Heterozygous
C6	M / 38	CKD with persistent proteinuria, hypertension, bilateral/complex renal cysts; Alport versus cystic kidney disease differential.	*COL4A5*	NM_033380.3	c.1871G>A	p.(Gly624Asp)	Missense / Hemizygous
C7	F / 25	Bilateral renal cystic disease, enlarged kidneys and bilateral angiomyolipomas; member of a previously reported *TSC2*/*PKD1* contiguous-deletion family [59]. The array interval was not interpreted as a sequence-level breakpoint.	*TSC2* / *PKD1*	*PKD1* / *TSC2* dosage locus	array-CGH: arr[GRCh37] 16p13.3(1980268_2147219)x1.	Not applicable	CNV deletion / Heterozygous *TSC2*/*PKD1* contiguous-gene deletion
C8	F / 28	Multisystemic von Hippel-Lindau phenotype with renal cysts, extrarenal involvement, and a positive family history.	*VHL*	NM_000551.4	c.233A>G	p.(Asn78Ser)	Missense / Heterozygous
C9-HF8992	F / 45	Familial hepatorenal polycystic disease; suspected ADPKD type 2 or minor ADPKD-spectrum gene.	*PKD2*	NM_000297.4	c.916C>T	p.(Arg306Ter)	Nonsense / Heterozygous
C10-P9	F / 45	ADPKD with bilateral renal and hepatic cysts, CKD G3b and positive family history.	*PKD1*	NM_001009944.3	c.7126C>T	p.(Gln2376Ter)	Nonsense / Heterozygous
C11-R13	F / 59	Familial hepatorenal polycystic disease with CKD and multiple affected relatives.	*PKD1*	NM_001009944.3	c.8311G>A	p.(Glu2771Lys)	Missense / Heterozygous
C12-WES7	M / 30	Early-onset CKD with small cystic kidneys, young-onset diabetes and family history suggestive of *HNF1B-related disease/ADTKD.*	*HNF1B*	NM_000458.4	c.1091C>G	p.(Ser364Ter)	Nonsense / Heterozygous
C13-EE299	M / 20	CKD G3aA3 with persistent hematuria, severe proteinuria, sensorineural hearing loss and maternal family history compatible with X-linked Alport syndrome.	*COL4A5*	NM_033380.3	c.4993A>T	p.(Ser1665Cys)	Missense / Hemizygous
C14-P56.2	F / 44	Birt-Hogg-Dubé/tuberous sclerosis differential due to facial cutaneous lesions; external negative-control case.	*FLCN* / *TSC1* / *TSC2*	Not applicable - no reportable variant	No pathogenic or likely pathogenic variant identified	Not applicable	No reportable variant / Not applicable
C15-R38	F / 39	Clinically definite tuberous sclerosis complex with renal cysts/angiomyolipomas and possible *TSC2*/*PKD1* contiguous-gene differential.	Not resolved	Not applicable	No conclusive result yet	Not applicable	Unresolved / Not applicable

Case	Sex / age	Clinical indication / phenotype	Gene	Transcript	Nucleotide change / CNV	Protein change	Variant type / zygosity
C16-R43	M / 20	Very-early-onset severe cystic kidney disease with childhood CKD progression to kidney failure; ARPKD/biallelic or digenic ADPKD/ciliopathy differential.	Not resolved	Not applicable	No conclusive result yet	Not applicable	Unresolved / Not applicable

Table S2B. Analytical support, final classification, and sample-level interpretation of the implementation samples

Case		VAF (%)	Total DP	REF/ALT	Final ACMG/AMP tier and evidence	Sample-level interpretation
C1	*PKD1* c.4072G>C, p.(Ala1358Pro)	70.59	17	5/12	VUS; PM2_Supporting	Panel-first VUS. Independently reidentified by short-read WES; not diagnostic. Segregation remains required. MLPA P351 negative.
C2	*PKD1* c.2180T>C, p.(Leu727Pro)	45.45	11	6/5	Pathogenic; PS4_VeryStrong + PP1_Supporting + PM2_Supporting + PM5_Supporting (11 points) [18,37–40,42,44–46,58]	Clinically informative, confirmation-dependent. The molecular category is conditional on authentic-*PKD1* attribution; validated locus-resolving confirmation is required.
C3	*PKD1* exon 1 heterozygous deletion	N/A	N/A	N/A	Pathogenic CNV	Pre-NGS MLPA-documented *PKD1* exon 1 deletion reference. Panel SNV/indel/read-depth analysis did not reliably resolve it. MLPA P351 positive.
C4	*PKD1* c.8369del, p.(Pro2790ArgfsTer85)	41.18	17	10/7	Likely Pathogenic; PVS1_VeryStrong + PM2_Supporting (9 points) [43]	Clinically informative, confirmation-dependent. Duplicated sequence and GGG-repeat context require validated *PKD1*-resolving confirmation. MLPA P351 negative.
C5	*PKD1* c.12682C>T, p.(Arg4228Ter)	38.89	18	11/7	Pathogenic; PVS1_Strong + PS4_Moderate + PP1_Moderate + PS3_Supporting + PM2_Supporting (10 points) [48–50]	Supported unique-locus observation. Independently reidentified by short-read WES; reproducible but not fully orthogonal. MLPA P351 negative.
C6	*COL4A5* c.1871G>A, p.(Gly624Asp)	100.00	1	0/1	Likely Pathogenic (external comparator)	Previously characterized comparator. Local DP 1 was insufficient for independent panel reporting; the observation was retained only for concordance and analytical-boundary assessment.
C7	*TSC2*/*PKD1* contiguous deletion	N/A	N/A	CNV signal	Pathogenic CNV	Pre-NGS contiguous *TSC2*/*PKD1* deletion reference. Panel read-depth behavior was concordant but not a standalone validated call. MLPA P351 positive. The biological classification derives from the pre-existing array-CGH record and the published family report [59]; it was not inferred from the panel read-depth signal.
C8	*VHL* c.233A>G, p.(Asn78Ser)	50.00	4	2/2	Likely Pathogenic (external comparator)	Previously characterized comparator. Local DP 4 was insufficient for independent panel reporting; the observation was retained only for concordance and analytical-boundary assessment.
C9-HF8992	*PKD2* c.916C>T, p.(Arg306Ter)	46.67	60	32/28	Pathogenic	Previously characterized comparator with adequate local support; the observation was retained only for concordance and was not counted as a panel-first finding.
C10-P9	*PKD1* c.7126C>T, p.(Gln2376Ter)	34.72	72	47/25	Pathogenic; PVS1_VeryStrong + PS4_Moderate + PM2_Supporting (11 points) [49,51,52]	Clinically informative, confirmation-dependent. Adequate nominal support does not establish authentic-*PKD1* attribution. MLPA P351 negative.
C11-R13	*PKD1* c.8311G>A, p.(Glu2771Lys)	51.39	360	175/185	Likely Pathogenic; PS4_Moderate + PP1_Moderate + PS3_Supporting + PM2_Supporting (6 points) [41,44,53–55]	Clinically informative, confirmation-dependent. Substantial local support does not replace validated locus-resolving confirmation.

Case		VAF (%)	Total DP	REF/ALT	Final ACMG/AMP tier and evidence	Sample-level interpretation
C12-WES7	*HNF1B c.1091C>G, p.(Ser364Ter)*	41.96	112	65/47	Likely Pathogenic; PVS1_VeryStrong + PM2_Supporting (9 points) [6]	Supported unique-locus observation with a clinically coherent *HNF1B* phenotype. Familial observations were not converted into PP1 without complete informative meioses.
C13-EE299	*COL4A5 c.4993A>T,* p.(Ser1665Cys)	100.00	18	0/18	Likely Pathogenic (external comparator)	Previously characterized external comparator. The biological classification derives from the prior diagnostic record and published report [22].
C14-P56.2	No reportable Pathogenic or Likely Pathogenic observation	N/A	N/A	N/A	No reportable Pathogenic or Likely Pathogenic variant	Predefined negative comparator: no reportable Pathogenic or Likely Pathogenic observation within the assessed context; not a universal molecular exclusion.
C15-R38	No conclusive finding	N/A	N/A	N/A	Unresolved	No conclusive finding. Negative result remains non-exclusionary; additional mechanism-specific testing required. MLPA P351 negative.
C16-R43	No conclusive finding	N/A	N/A	N/A	Unresolved	No conclusive finding. Severe early-onset phenotype supports broader and/or long-read follow-up. MLPA P351 negative.

Note. Reference numbers correspond to the bibliography of the main manuscript. References appended to the evidence column identify the principal exact-allele, recurrence, segregation, variant-specific functional, and gene-disease/phenotypic-context sources reviewed. Clinical database classifications were used for traceability and contextual concordance and were not counted as independent ACMG/AMP evidence. Population rarity was assessed in gnomAD v4.1.0 [47]. General criterion definitions and evidence strengths follow the framework described in Supplementary Methods and Section 2.7 of the main manuscript [28–36].

Table S2C. Case-level interpretability summary

Case	Finding	Final tier/status	Study interpretation
C1	*PKD1* c.4072G>C, p.(Ala1358Pro)	VUS	Panel-first VUS; independently reidentified by short-read WES; not diagnostic. MLPA P351 negative.
C2	*PKD1* c.2180T>C, p.(Leu727Pro)	Pathogenic	Confirmation-dependent duplicated-region observation; validated *PKD1*-resolving confirmation required before definitive sample-level reporting.
C3	*PKD1* exon 1 heterozygous deletion	Pathogenic CNV	Pre-NGS MLPA-documented CNV reference; panel did not reliably resolve the exon 1 event. The biological classification was established by the pre-existing diagnostic MLPA P351 result.
C4	*PKD1* c.8369del, p.(Pro2790ArgfsTer85)	Likely Pathogenic	Confirmation-dependent duplicated-region frameshift observation; validated *PKD1*-resolving confirmation required.
C5	*PKD1* c.12682C>T, p.(Arg4228Ter)	Pathogenic	Supported unique-locus observation; independently reidentified by short-read WES.
C6	*COL4A5* c.1871G>A, p.(Gly624Asp)	Externally diagnostic comparator	Previously characterized comparator; local DP 1 was insufficient for independent reporting.
C7	*TSC2*/*PKD1* contiguous deletion	Pathogenic CNV	Pre-NGS MLPA/array-documented CNV reference; panel read-depth behavior was concordant only. The biological classification derives from the pre-existing MLPA/array-CGH record and the published family report [59].
C8	*VHL* c.233A>G, p.(Asn78Ser)	Externally diagnostic comparator	Previously characterized comparator; local DP 4 was insufficient for independent reporting.
C9-HF8992	*PKD2* c.916C>T, p.(Arg306Ter)	Pathogenic	Previously characterized comparator with adequate local support and concordant observation
C10-P9	*PKD1* c.7126C>T, p.(Gln2376Ter)	Pathogenic	Confirmation-dependent duplicated-region observation; validated locus-resolving confirmation required.

Case	Finding	Final tier/status	Study interpretation
C11-R13	*PKD1* c.8311G>A, p.(Glu2771Lys)	Likely Pathogenic	Confirmation-dependent despite strong nominal support and familial concordance; validated locus-resolving confirmation remains required.
C12-WES7	*HNF1B* c.1091C>G, p.(Ser364Ter)	Likely Pathogenic	Supported unique-locus observation with a clinically coherent *HNF1B phenotype.*
C13-EE299	*COL4A5* c.4993A>T, p.(Ser1665Cys)	Likely Pathogenic (external comparator)	Previously characterized comparator; not counted as a panel-first finding.
C14-P56.2	No reportable Pathogenic or Likely Pathogenic observation	Negative comparator	Negative comparator within the assessed context; not a universal molecular exclusion.
C15-R38	No conclusive finding	Unresolved	Negative result remains non-exclusionary; additional testing required.
C16-R43	No conclusive finding	Unresolved	Negative result remains non-exclusionary; broader and/or long-read follow-up indicated.

Table S2D. Retrospective site-specific locus-aware FASTQ reassessment of four clinically informative duplicated-region *PKD1* observations. Strict fragment counts are reported in REF/ALT order, use a different recovery denominator from nominal DRAGEN DP, and do not establish global callability across exons 1–33. C1 was not included because its sequence change remained a non-diagnostic VUS and was not part of the clinically informative duplicated-region reassessment.

Case	Variant	Nominal DP	Nominal REF/ALT	VAF	Strict locus-restricted REF/ALT fragments	locus-support grade	Interpretation
C2	*PKD1* c.2180T>C, p.(Leu727Pro)	11	6/5	45.45%	1/3	Limited;	Sparse site-specific support; different denominator from nominal DP; confirmation-dependent.
C4	*PKD1* c.8369del, p.(Pro2790ArgfsTer85)	17	10/7	41.18%	6/6	Moderate;	Balanced recovered fragments; repeat context and duplicated locus remain confirmation-dependent.
C10-P9	*PKD1* c.7126C>T, p.(Gln2376Ter)	72	47/25	34.72%	15/8	Moderate;	Site-specific support present; authentic-locus attribution not prospectively validated.
C11-R13	*PKD1* c.8311G>A, p.(Glu2771Lys)	360	175/185	51.39%	217/225	Strong.	Substantial site-specific computational support; not a validated locus-resolving clinical result.

Coverage Summaries

Tables S3–S13 were calculated from the DRAGEN FullCoverage_GenePanel outputs generated for the 16 study samples using the same fixed denominator of 694 analytical target intervals per sample. Table S6A was generated from gene-summary records in Supplementary Data File S5. The tables distinguish interval mean depth, true interval minimum depth, base-level completeness, and descriptive gene-average aligned depth.

Table S6A reports the unweighted arithmetic mean of DRAGEN interval Mean Coverage values assigned to each gene and sample.

Table S3. Run-specific arithmetic-mean and pooled depth-completeness summary across the fixed 694-record denominator. Because each run contained eight samples with the same denominator, each pooled sample–interval proportion equals the arithmetic mean of the eight per-sample proportions.

Metric	Run 1	Run 2	Interpretation
Samples, n	8	8	Same number of samples; different samples and analytical roles
Length-weighted mean interval depth	11.6×	145.5×	Length-weighted from interval mean depth
Interval records with mean depth ≥10×	2,335/5,552 (42.1%)	4,775/5,552 (86.0%)	Record-level mean-depth descriptor

Metric	Run 1	Run 2	Interpretation
Interval records with mean depth ≥20×	463/5,552 (8.3%)	4,592/5,552 (82.7%)	Descriptive implementation benchmark
Interval records with mean depth ≥30×	54/5,552 (1.0%)	4,374/5,552 (78.8%)	More conservative record-level descriptor
Interval records with minimum depth ≥10×	1,513/5,552 (27.3%)	4,555/5,552 (82.0%)	Stricter focal-depth descriptor
Interval records with minimum depth ≥20×	158/5,552 (2.8%)	4,256/5,552 (76.7%)	Stricter whole-record criterion
Interval-record bases covered at ≥10×	437,190/824,600 (53.0%)	739,087/824,600 (89.6%)	Base-level completeness
Intervals below 20× mean depth/sample	636.1/694	120.0/694	Complement of mean-depth criterion
Intervals below 20× minimum depth/sample	674.2/694	162.0/694	Complement of minimum-depth criterion

Table S4. Per-sample proportions of the fixed 694 analytical target-interval records meeting interval-mean and interval-minimum ≥20× criteria, with base-level ≥10× completeness.

Case/Sample	Run	Length-weighted mean depth	Intervals mean ≥20×	Intervals minimum ≥20×	Interval bases ≥10×
C1	Run 1	11.65×	5.33%	1.01%	58.66%
C2	Run 1	10.52×	2.59%	0.58%	50.49%
C3	Run 1	15.77×	18.30%	7.20%	72.67%
C4	Run 1	17.06×	24.06%	9.65%	75.72%
C5	Run 1	13.62×	10.52%	2.88%	68.88%
C6	Run 1	6.53×	0.00%	0.00%	23.11%
C7	Run 1	12.55×	5.91%	1.44%	66.32%
C8	Run 1	5.16×	0.00%	0.00%	8.28%
C9-HF8992	Run 2	137.62×	81.70%	75.22%	89.12%
C10-P9	Run 2	113.34×	80.98%	74.35%	88.95%
C11-R13	Run 2	163.31×	83.57%	78.53%	90.16%
C12-WES7	Run 2	158.09×	82.85%	76.95%	89.44%
C13-EE299	Run 2	171.91×	83.14%	76.80%	89.95%
C14-P56.2	Run 2	135.00×	83.43%	77.81%	89.95%
C15-R38	Run 2	155.14×	84.58%	79.25%	90.01%
C16-R43	Run 2	129.80×	81.41%	74.35%	89.46%

Table S5. Per-sample depth completeness for *PKD1*, *PKD2*, *DNAJB11*, and the *PKD1* exon 1 interval. Gene columns report the percentage of gene-assigned analytical intervals meeting each criterion.

Case/Sample	Run	*PKD1 mean ≥20× (%)*	*PKD1 minimum ≥20× (%)*	*PKD2 mean ≥20× (%)*	*PKD2 minimum ≥20× (%)*	*DNAJB11 mean ≥20× (%)*	*DNAJB11 minimum ≥20× (%)*	*PKD1 exon 1 mean (×)*	*PKD1 exon 1 minimum (×)*
C1	Run 1	8.70	0.00	26.67	6.67	20.00	10.00	0.63	0
C2	Run 1	4.35	2.17	6.67	6.67	0.00	0.00	0.56	0
C3	Run 1	41.30	21.74	53.33	26.67	0.00	0.00	0.61	0

Case/Sample	Run	*PKD1 mean ≥20× (%)*	*PKD1 minimum ≥20× (%)*	*PKD2 mean ≥20× (%)*	*PKD2 minimum ≥20× (%)*	*DNAJB11 mean ≥20× (%)*	*DNAJB11 minimum ≥20× (%)*	*PKD1 exon 1 mean (×)*	*PKD1 exon 1 minimum (×)*
C4	Run 1	45.65	17.39	46.67	40.00	0.00	0.00	1.27	0
C5	Run 1	15.22	4.35	26.67	0.00	0.00	0.00	0.00	0
C6	Run 1	0.00	0.00	0.00	0.00	0.00	0.00	0.00	0
C7	Run 1	2.17	0.00	46.67	13.33	10.00	0.00	0.47	0
C8	Run 1	0.00	0.00	0.00	0.00	0.00	0.00	0.00	0
C9-HF8992	Run 2	97.83	97.83	53.33	33.33	60.00	50.00	7.33	0
C10-P9	Run 2	97.83	97.83	53.33	33.33	60.00	40.00	8.00	0
C11-R13	Run 2	97.83	97.83	53.33	40.00	60.00	50.00	6.44	0
C12-WES7	Run 2	97.83	97.83	53.33	33.33	60.00	50.00	2.35	0
C13-EE299	Run 2	97.83	97.83	53.33	33.33	60.00	50.00	5.50	0
C14-P56.2	Run 2	97.83	97.83	53.33	33.33	60.00	50.00	4.88	0
C15-R38	Run 2	97.83	97.83	53.33	40.00	60.00	50.00	5.97	0
C16-R43	Run 2	97.83	97.83	53.33	33.33	60.00	50.00	11.59	0

Table S6. Across-sample mean proportion of gene-assigned analytical target intervals meeting mean-depth ≥20× and minimum-depth ≥20× criteria by configuration, with Run 2 base-level ≥10× completeness.

Gene	Records	Run 1 mean ≥20× (%)	Run 1 minimum ≥20× (%)	Run 2 mean ≥20× (%)	Run 2 minimum ≥20× (%)	Run 2 bases ≥10× (%)
UMOD	13	14.4	2.9	100.0	100.0	100.0
TSC2	45	24.2	15.3	100.0	100.0	100.0
REN	10	5.0	0.0	100.0	100.0	100.0
PRKCSH	16	5.5	1.6	100.0	100.0	100.0
GANAB	25	14.0	7.0	100.0	100.0	100.0
FLCN	13	6.7	1.0	100.0	100.0	100.0
HNF1B	10	10.0	2.5	100.0	98.8	100.0
SEC61A1	13	5.8	1.9	100.0	87.5	99.6
VHL	4	0.0	0.0	100.0	75.0	100.0
PKD1	46	14.7	5.7	97.8	97.8	98.9
NOTCH2	35	15.4	1.1	97.1	90.4	98.8
LRP5	24	11.5	1.0	95.8	95.8	99.1
DZIP1L	16	3.1	0.8	93.8	93.8	93.0
COL4A1	53	1.9	0.7	93.4	93.2	98.3
COL4A3	52	2.9	0.5	90.1	88.5	95.6
SEC61B	4	12.5	0.0	87.5	84.4	100.0
COL4A4	47	2.7	0.8	87.2	87.0	94.7
COL4A5	53	0.0	0.0	85.4	79.5	98.3

Gene	Records	Run 1 mean ≥20× (%)	Run 1 minimum ≥20× (%)	Run 2 mean ≥20× (%)	Run 2 minimum ≥20× (%)	Run 2 bases ≥10× (%)
TSC1	21	32.1	15.5	84.5	72.0	95.0
MUC1	12	6.2	1.0	81.2	50.0	78.6
PKHD1	67	6.3	0.2	73.3	58.2	84.9
ALG9	18	2.8	0.7	61.8	42.4	78.9
DNAJB11	10	3.8	1.2	60.0	48.8	61.5
PKD2	15	25.8	11.7	53.3	35.0	68.3
ALG8	18	3.5	0.0	37.5	17.4	50.1
OFD1	23	1.1	0.0	34.8	17.4	51.0
SEC63	21	1.8	0.0	17.9	16.1	28.5
ALG5	10	2.5	0.0	16.2	10.0	25.6

Table S6A. Run 2 descriptive gene-level aligned-depth matrix by sample: unweighted arithmetic mean of DRAGEN interval Mean Coverage values across all analytical target intervals assigned to each gene.

Gene	C9-HF8992	C10-P9	C11-R13	C12-WES7	C13-EE299	C14-P56.2	C15-R38	C16-R43	Across-sample mean
PKD1	261.95	207.46	313.62	292.85	327.99	245.42	263.58	247.25	270.01
PKD2	48.96	46.35	58.99	65.52	66.38	52.85	63.21	51.76	56.75
DNAJB11	47.10	38.78	52.02	63.29	66.35	48.32	60.53	47.86	53.03
GANAB	143.02	120.32	187.65	177.73	218.25	147.72	175.10	141.59	163.92
HNF1B	184.36	140.90	203.32	209.19	197.51	170.56	212.42	164.04	185.29
UMOD	154.01	127.92	188.41	187.89	186.20	157.01	186.21	147.12	166.85
TSC2	244.67	198.53	302.69	285.98	323.11	234.01	252.22	229.14	258.80
PRKCSH	196.45	157.32	230.22	223.24	268.97	191.97	229.38	177.74	209.41
ALG5	19.71	16.16	21.78	24.61	31.68	18.36	28.07	22.03	22.80
ALG8	15.28	13.66	20.31	20.68	20.42	16.28	19.51	14.60	17.59
ALG9	27.20	25.40	36.43	36.93	36.13	29.75	36.34	29.75	32.24
PKHD1	52.39	46.28	65.11	70.11	72.64	58.12	64.69	55.68	60.63
DZIP1L	135.81	110.38	157.06	160.63	165.63	129.51	158.35	126.38	142.97
TSC1	68.09	60.09	85.90	89.08	91.84	74.00	84.45	68.72	77.77
COL4A1	104.13	91.60	129.71	132.65	134.44	109.41	127.30	106.69	116.99
REN	139.06	113.99	171.56	170.39	169.41	141.14	164.37	135.06	150.62
SEC61A1	99.20	86.85	117.09	119.88	130.04	100.93	123.94	102.75	110.09
SEC63	11.41	11.64	13.80	15.68	17.09	11.07	16.90	13.37	13.87
LRP5	230.71	182.67	267.27	255.20	267.48	216.16	266.35	209.93	236.97
SEC61B	131.37	109.27	145.21	143.86	149.80	111.34	139.35	121.40	131.45
VHL	159.79	110.64	165.97	164.75	200.24	132.38	182.66	139.86	157.04
COL4A3	71.68	63.43	83.75	92.06	91.61	78.78	87.71	75.93	80.62
COL4A4	82.40	72.93	99.59	104.82	108.17	89.02	101.82	82.80	92.69
COL4A5	64.29	56.70	70.47	45.30	51.73	73.19	82.23	35.86	59.97

Gene	C9-HF8992	C10-P9	C11-R13	C12-WES7	C13-EE299	C14-P56.2	C15-R38	C16-R43	Across-sample mean
MUC1	110.59	98.29	131.06	118.06	132.38	106.58	128.31	103.56	116.11
FLCN	190.88	153.75	233.62	213.48	228.20	185.04	230.33	175.28	201.32
NOTCH2	111.99	94.21	131.46	141.62	145.41	115.25	136.54	109.84	123.29
OFD1	30.26	25.16	36.44	19.26	20.72	30.45	39.13	15.69	27.14

Values are expressed in ×. This metric is distinct from interval minimum depth, the proportion of intervals or bases reaching a threshold, site-level DP, and authentic-locus callability. A high gene-average value can coexist with a focal weak interval.

Table S7. Run 1 sample-by-gene percentage of gene-assigned analytical target intervals with interval mean depth ≥20×.

Gene	C1	C2	C3	C4	C5	C6	C7	C8
PKD1	8.7	4.3	41.3	45.7	15.2	0.0	2.2	0.0
PKD2	26.7	6.7	53.3	46.7	26.7	0.0	46.7	0.0
DNAJB11	20.0	0.0	0.0	0.0	0.0	0.0	10.0	0.0
GANAB	0.0	0.0	36.0	44.0	24.0	0.0	8.0	0.0
HNF1B	0.0	0.0	10.0	40.0	10.0	0.0	20.0	0.0
UMOD	7.7	15.4	23.1	46.2	23.1	0.0	0.0	0.0
TSC2	13.3	11.1	68.9	68.9	31.1	0.0	0.0	0.0
PRKCSH	0.0	0.0	12.5	18.8	6.2	0.0	6.2	0.0
ALG5	10.0	0.0	0.0	0.0	0.0	0.0	10.0	0.0
ALG8	0.0	0.0	0.0	5.6	5.6	0.0	16.7	0.0
ALG9	0.0	0.0	11.1	5.6	0.0	0.0	5.6	0.0
PKHD1	7.5	3.0	9.0	19.4	7.5	0.0	4.5	0.0
DZIP1L	0.0	0.0	6.2	18.8	0.0	0.0	0.0	0.0
TSC1	33.3	9.5	66.7	61.9	42.9	0.0	42.9	0.0
COL4A1	0.0	0.0	0.0	7.5	5.7	0.0	1.9	0.0
REN	0.0	0.0	0.0	30.0	10.0	0.0	0.0	0.0
SEC61A1	0.0	0.0	0.0	15.4	15.4	0.0	15.4	0.0
SEC63	9.5	0.0	0.0	0.0	0.0	0.0	4.8	0.0
LRP5	4.2	4.2	20.8	41.7	8.3	0.0	12.5	0.0
SEC61B	0.0	0.0	25.0	25.0	25.0	0.0	25.0	0.0
VHL	0.0	0.0	0.0	0.0	0.0	0.0	0.0	0.0
COL4A3	1.9	1.9	1.9	11.5	5.8	0.0	0.0	0.0
COL4A4	2.1	2.1	6.4	8.5	2.1	0.0	0.0	0.0
COL4A5	0.0	0.0	0.0	0.0	0.0	0.0	0.0	0.0
MUC1	0.0	0.0	25.0	25.0	0.0	0.0	0.0	0.0
FLCN	0.0	0.0	23.1	23.1	7.7	0.0	0.0	0.0
NOTCH2	5.7	2.9	40.0	48.6	22.9	0.0	2.9	0.0
OFD1	0.0	0.0	4.3	0.0	0.0	0.0	4.3	0.0

Table S8. Run 1 sample-by-gene percentage of gene-assigned analytical target intervals with true interval minimum depth ≥20×.

Gene	C1	C2	C3	C4	C5	C6	C7	C8
PKD1	0.0	2.2	21.7	17.4	4.3	0.0	0.0	0.0
PKD2	6.7	6.7	26.7	40.0	0.0	0.0	13.3	0.0
DNAJB11	10.0	0.0	0.0	0.0	0.0	0.0	0.0	0.0
GANAB	0.0	0.0	20.0	24.0	8.0	0.0	4.0	0.0
HNF1B	0.0	0.0	0.0	20.0	0.0	0.0	0.0	0.0
UMOD	0.0	0.0	0.0	23.1	0.0	0.0	0.0	0.0
TSC2	4.4	2.2	48.9	48.9	17.8	0.0	0.0	0.0
PRKCSH	0.0	0.0	6.2	6.2	0.0	0.0	0.0	0.0
ALG5	0.0	0.0	0.0	0.0	0.0	0.0	0.0	0.0
ALG8	0.0	0.0	0.0	0.0	0.0	0.0	0.0	0.0
ALG9	0.0	0.0	0.0	5.6	0.0	0.0	0.0	0.0
PKHD1	0.0	0.0	0.0	0.0	0.0	0.0	1.5	0.0
DZIP1L	0.0	0.0	0.0	6.2	0.0	0.0	0.0	0.0
TSC1	9.5	4.8	28.6	38.1	23.8	0.0	19.0	0.0
COL4A1	0.0	0.0	0.0	1.9	1.9	0.0	1.9	0.0
REN	0.0	0.0	0.0	0.0	0.0	0.0	0.0	0.0
SEC61A1	0.0	0.0	0.0	0.0	7.7	0.0	7.7	0.0
SEC63	0.0	0.0	0.0	0.0	0.0	0.0	0.0	0.0
LRP5	0.0	0.0	0.0	8.3	0.0	0.0	0.0	0.0
SEC61B	0.0	0.0	0.0	0.0	0.0	0.0	0.0	0.0
VHL	0.0	0.0	0.0	0.0	0.0	0.0	0.0	0.0
COL4A3	1.9	0.0	0.0	0.0	1.9	0.0	0.0	0.0
COL4A4	0.0	0.0	2.1	4.3	0.0	0.0	0.0	0.0
COL4A5	0.0	0.0	0.0	0.0	0.0	0.0	0.0	0.0
MUC1	0.0	0.0	0.0	8.3	0.0	0.0	0.0	0.0
FLCN	0.0	0.0	0.0	7.7	0.0	0.0	0.0	0.0
NOTCH2	0.0	0.0	2.9	5.7	0.0	0.0	0.0	0.0
OFD1	0.0	0.0	0.0	0.0	0.0	0.0	0.0	0.0

Table S9. Run 2 sample-by-gene percentage of gene-assigned analytical target intervals with interval mean depth ≥20×.

**Gene**	**C9**	**C10**	**C11**	**C12**	**C13**	**C14**	**C15**	**C16**	**Across-sample mean**
PKD1	97.8	97.8	97.8	97.8	97.8	97.8	97.8	97.8	97.8
PKD2	53.3	53.3	53.3	53.3	53.3	53.3	53.3	53.3	53.3

Gene	C9	C10	C11	C12	C13	C14	C15	C16	Across-sample mean
DNAJB11	60.0	60.0	60.0	60.0	60.0	60.0	60.0	60.0	60.0
GANAB	100.0	100.0	100.0	100.0	100.0	100.0	100.0	100.0	100.0
HNF1B	100.0	100.0	100.0	100.0	100.0	100.0	100.0	100.0	100.0
UMOD	100.0	100.0	100.0	100.0	100.0	100.0	100.0	100.0	100.0
TSC2	100.0	100.0	100.0	100.0	100.0	100.0	100.0	100.0	100.0
PRKCSH	100.0	100.0	100.0	100.0	100.0	100.0	100.0	100.0	100.0
ALG5	10.0	10.0	20.0	20.0	20.0	20.0	20.0	10.0	16.2
ALG8	38.9	33.3	38.9	38.9	38.9	38.9	38.9	33.3	37.5
ALG9	55.6	44.4	61.1	66.7	66.7	61.1	72.2	66.7	61.8
PKHD1	68.7	68.7	76.1	76.1	74.6	73.1	74.6	74.6	73.3
DZIP1L	93.8	93.8	93.8	93.8	93.8	93.8	93.8	93.8	93.8
TSC1	81.0	81.0	85.7	85.7	85.7	85.7	85.7	85.7	84.5
COL4A1	92.5	92.5	94.3	94.3	92.5	92.5	94.3	94.3	93.4
REN	100.0	100.0	100.0	100.0	100.0	100.0	100.0	100.0	100.0
SEC61A1	100.0	100.0	100.0	100.0	100.0	100.0	100.0	100.0	100.0
SEC63	14.3	19.0	19.0	19.0	19.0	14.3	19.0	19.0	17.9
LRP5	95.8	95.8	95.8	95.8	95.8	95.8	95.8	95.8	95.8
SEC61B	75.0	75.0	75.0	100.0	100.0	100.0	100.0	75.0	87.5
VHL	100.0	100.0	100.0	100.0	100.0	100.0	100.0	100.0	100.0
COL4A3	88.5	90.4	90.4	88.5	90.4	90.4	92.3	90.4	90.1
COL4A4	85.1	83.0	87.2	89.4	89.4	89.4	89.4	85.1	87.2
COL4A5	86.8	84.9	88.7	81.1	84.9	90.6	94.3	71.7	85.4
MUC1	83.3	83.3	83.3	75.0	83.3	83.3	75.0	83.3	81.2
FLCN	100.0	100.0	100.0	100.0	100.0	100.0	100.0	100.0	100.0
NOTCH2	97.1	97.1	97.1	97.1	97.1	97.1	97.1	97.1	97.1
OFD1	39.1	30.4	39.1	30.4	30.4	39.1	43.5	26.1	34.8

Table S10. Run 2 sample-by-gene percentage of gene-assigned analytical target intervals with true interval minimum depth ≥20×.

Gene	C9	C10	C11	C12	C13	C14	C15	C16	Across-sample mean
PKD1	97.8	97.8	97.8	97.8	97.8	97.8	97.8	97.8	97.8
PKD2	33.3	33.3	40.0	33.3	33.3	33.3	40.0	33.3	35.0
DNAJB11	50.0	40.0	50.0	50.0	50.0	50.0	50.0	50.0	48.8
GANAB	100.0	100.0	100.0	100.0	100.0	100.0	100.0	100.0	100.0
HNF1B	90.0	100.0	100.0	100.0	100.0	100.0	100.0	100.0	98.8
UMOD	100.0	100.0	100.0	100.0	100.0	100.0	100.0	100.0	100.0

Gene	C9	C10	C11	C12	C13	C14	C15	C16	Across-sample mean
TSC2	100.0	100.0	100.0	100.0	100.0	100.0	100.0	100.0	100.0
PRKCSH	100.0	100.0	100.0	100.0	100.0	100.0	100.0	100.0	100.0
ALG5	10.0	10.0	10.0	10.0	10.0	10.0	10.0	10.0	10.0
ALG8	11.1	11.1	22.2	22.2	22.2	22.2	22.2	5.6	17.4
ALG9	38.9	22.2	50.0	50.0	50.0	38.9	44.4	44.4	42.4
PKHD1	55.2	49.3	64.2	62.7	59.7	59.7	56.7	58.2	58.2
DZIP1L	93.8	93.8	93.8	93.8	93.8	93.8	93.8	93.8	93.8
TSC1	66.7	66.7	71.4	76.2	71.4	76.2	76.2	71.4	72.0
COL4A1	92.5	92.5	94.3	94.3	90.6	92.5	94.3	94.3	93.2
REN	100.0	100.0	100.0	100.0	100.0	100.0	100.0	100.0	100.0
SEC61A1	76.9	84.6	92.3	92.3	92.3	84.6	92.3	84.6	87.5
SEC63	14.3	14.3	14.3	14.3	19.0	14.3	19.0	19.0	16.1
LRP5	95.8	95.8	95.8	95.8	95.8	95.8	95.8	95.8	95.8
SEC61B	75.0	75.0	75.0	100.0	100.0	75.0	100.0	75.0	84.4
VHL	75.0	75.0	75.0	75.0	75.0	75.0	75.0	75.0	75.0
COL4A3	84.6	88.5	90.4	86.5	88.5	88.5	90.4	90.4	88.5
COL4A4	85.1	83.0	85.1	89.4	89.4	89.4	89.4	85.1	87.0
COL4A5	86.8	81.1	86.8	69.8	73.6	90.6	94.3	52.8	79.5
MUC1	50.0	50.0	50.0	50.0	50.0	50.0	50.0	50.0	50.0
FLCN	100.0	100.0	100.0	100.0	100.0	100.0	100.0	100.0	100.0
NOTCH2	82.9	88.6	91.4	91.4	91.4	91.4	94.3	91.4	90.4
OFD1	17.4	17.4	21.7	13.0	13.0	17.4	26.1	13.0	17.4

Table S11. Ten *PKD1* analytical target intervals with the lowest Run 2 across-sample mean interval depth, with the corresponding true interval-minimum values. Only exon 1 failed the ≥20× criteria.

PKD1 interval (GRCh38)	Exon proxy	Mean of interval means	Range of interval means	Mean of true interval minima	Range of true interval minima	Samples ≥20× (mean; minimum)	Interpretation
chr16:2135474-2135689	Exon 1	6.5×	2.35-11.59	0.0×	0.00-0.00	mean 0/8; minimum 0/8	Principal recurrent weak interval; true minimum depth was 0x in all Run 2 samples.
chr16:2115991-2116118	Exon 9	85.0×	68.88-111.11	52.4×	42.00-77.00	mean 8/8; minimum 8/8	Mean-depth benchmark met in all Run 2 samples; minimum-depth behavior reported separately.
chr16:2091422-2091597	Exon 42	110.0×	83.73-130.81	49.0×	29.00-65.00	mean 8/8; minimum 8/8	Mean-depth benchmark met in all Run 2 samples; minimum-depth behavior reported separately.
chr16:2119113-2119185	Exon 3	125.8×	90.86-160.59	106.1×	69.00-139.00	mean 8/8; minimum 8/8	Mean-depth benchmark met in all Run 2 samples; minimum-depth behavior reported separately.
chr16:2104497-2104642	Exon 22	165.9×	135.38-198.11	116.4×	88.00-146.00	mean 8/8; minimum 8/8	Mean-depth benchmark met in all Run 2 samples; minimum-depth behavior reported separately.

PKD1 interval (GRCh38)	Exon proxy	Mean of interval means	Range of interval means	Mean of true interval minima	Range of true interval minima	Samples ≥20× (mean; minimum)	Interpretation
chr16:2113160-2113292	Exon 12	178.6×	125.72-227.26	59.8×	51.00-80.00	mean 8/8; minimum 8/8	Mean-depth benchmark met in all Run 2 samples; minimum-depth behavior reported separately.
chr16:2106804-2106948	Not assigned	190.1×	57.03-264.54	163.8×	31.00-228.00	mean 8/8; minimum 8/8	Mean-depth benchmark met in all Run 2 samples; minimum-depth behavior reported separately.
chr16:2090883-2091174	Not assigned	196.4×	169.15-227.75	136.2×	98.00-152.00	mean 8/8; minimum 8/8	Mean-depth benchmark met in all Run 2 samples; minimum-depth behavior reported separately.
chr16:2105321-2105474	Not assigned	224.7×	175.71-290.36	185.8×	134.00-244.00	mean 8/8; minimum 8/8	Mean-depth benchmark met in all Run 2 samples; minimum-depth behavior reported separately.
chr16:2097727-2097780	Not assigned	228.2×	179.44-287.59	216.9×	163.00-280.00	mean 8/8; minimum 8/8	Mean-depth benchmark met in all Run 2 samples; minimum-depth behavior reported separately.

Table S12. Global DRAGEN mapping/alignment quality-control descriptors per sample. These metrics are not substitutes for target-specific depth or locus-specific callability.

Sample	Run	Total reads	Q30 bases (%)	Mapped reads (%)	Duplicate reads (%)	Properly paired (%)	Multi-mapping (%)	MAPQ >=40 (%)	Insert mean (bp)	Insert median (bp)
C1	Run 1	161,602	85.72	98.06	1.33	96.84	10.06	83.21	242.56	213
C2	Run 1	231,644	89.95	98.53	1.11	98.03	7.38	87.34	244.47	215
C3	Run 1	166,452	88.16	98.65	1.24	98.04	11.16	82.17	253.93	225
C4	Run 1	159,148	87.05	98.79	1.02	98.12	15.37	76.69	218.59	195
C5	Run 1	168,202	89.72	98.86	0.98	98.40	10.71	83.14	205.91	182
C6	Run 1	127,534	89.56	98.61	0.97	98.11	10.88	82.89	204.18	184
C7	Run 1	261,164	89.83	98.67	1.01	98.16	8.47	86.60	176.70	162
C8	Run 1	170,714	90.18	98.59	0.83	98.15	7.05	88.37	168.31	151
C9-HF8992	Run 2	1,155,542	92.58	97.99	4.22	97.72	30.73	58.58	151.81	146
C10-P9	Run 2	859,420	92.21	97.92	4.07	97.55	26.03	63.96	158.23	152
C11-R13	Run 2	1,191,474	92.73	97.97	4.54	97.61	25.91	63.90	164.07	158
C12-WES7	Run 2	1,265,218	92.69	98.01	4.67	97.67	29.26	59.83	161.28	156
C13-EE299	Run 2	1,484,722	92.42	98.00	5.31	97.82	31.83	57.46	154.14	147
C14-P56.2	Run 2	1,059,182	92.16	98.23	4.58	97.84	29.01	60.35	163.31	157
C15-R38	Run 2	1,388,158	92.35	98.23	5.26	97.94	33.61	55.66	155.21	150
C16-R43	Run 2	1,012,138	91.68	97.97	4.45	97.63	26.65	63.86	155.13	150

Table S13. Complete 28-gene Run 2 aligned-depth completeness summary: across-sample mean proportions for interval mean ≥20×, interval minimum ≥20×, and interval bases ≥10×.

The three depth-completeness fields in Table S13 are complementary: the interval-mean proportion summarizes average record depth, the interval-minimum proportion detects focal within-record weakness, and the base-level proportion quantifies bases covered at ≥10×. None of these aligned-depth metrics establishes authentic-locus attribution in duplicated *PKD1* sequence or assessment of all molecular mechanisms.

Gene	Records	Run 2 mean depth ≥20×	Run 2 minimum depth ≥20×	Run 2 bases ≥10× / note
UMOD	13	100.0%	100.0%	100.0%
TSC2	45	100.0%	100.0%	100.0%
REN	10	100.0%	100.0%	100.0%
PRKCSH	16	100.0%	100.0%	100.0%
GANAB	25	100.0%	100.0%	100.0%
FLCN	13	100.0%	100.0%	100.0%
HNF1B	10	100.0%	98.8%	100.0%
SEC61A1	13	100.0%	87.5%	99.6%
VHL	4	100.0%	75.0%	100.0%
PKD1	46	97.8%	97.8%	98.9%; exon 1 weak; duplicated sequence unresolved
NOTCH2	35	97.1%	90.4%	98.8%
LRP5	24	95.8%	95.8%	99.1%
DZIP1L	16	93.8%	93.8%	93.0%
COL4A1	53	93.4%	93.2%	98.3%
COL4A3	52	90.1%	88.5%	95.6%
SEC61B	4	87.5%	84.4%	100.0%
COL4A4	47	87.2%	87.0%	94.7%
COL4A5	53	85.4%	79.5%	98.3%
TSC1	21	84.5%	72.0%	95.0%
MUC1	12	81.2%	50.0%	78.6%; VNTR mechanism not assessed
PKHD1	67	73.3%	58.2%	84.9%
ALG9	18	61.8%	42.4%	78.9%
DNAJB11	10	60.0%	48.8%	61.5%
PKD2	15	53.3%	35.0%	68.3%
ALG8	18	37.5%	17.4%	50.1%
OFD1	23	34.8%	17.4%	51.0%
SEC63	21	17.9%	16.1%	28.5%
ALG5	10	16.2%	10.0%	25.6%

Supplementary Notes

Mapped-read percentage is not an on-target metric. A record may have a satisfactory interval mean while containing one or more focal low-depth bases; interval mean depth, interval minimum depth, base-level completeness, and site-level DP must therefore remain separate. The *MUC1 coding VNTR and authentic-locus attribution in duplicated PKD1 sequence are not clinically excluded by these depth summaries.*

Run 2 provided the primary higher-output dataset for broad depth characterization. Its profile cannot be attributed to pre-capture pooling, sequencing output, reagent configuration, or another single variable because multiple factors and sample composition changed concurrently. The shared probe design permitted descriptive assessment of recurrent interval-specific patterns, including persistent *PKD1 exon 1* undercoverage.

References cited in the Supplementary Materials correspond to the numbered reference list in the main manuscript.

Supplementary Data Files S1–S5

Supplementary Code S1
