# Supplementary material for: From Guidelines to Practice: Pilot Implementation and Analytical Boundaries of a Focused ADPKD-Spectrum Gene Panel
Source: Diagnostics (Basel). 2026 Aug 28;16(17):2770. doi: 10.3390/diagnostics16172770 (PMC13564536; doi:10.3390/diagnostics16172770)
Supplement: Supplementary file 1 [file diagnostics-16-02770-s001.zip › Supplementary_Protocol_S1_SureSelect_XT_HS2.pdf]

Supplementary Protocol S1

SureSelect XT HS2 DNA library preparation, custom hybrid capture, and MiSeq sequencing for the focused 28-gene ADPKD-spectrum panel

Scope. This protocol records the wet-lab procedure used for two independent eight-sample sequencing runs. Both configurations used the same custom Tier 1 biotinylated RNA-probe design and the same SureSelect XT HS2 chemistry. Run 1 used individual hybridization and capture followed by post-capture pooling. Run 2 used normalized pre-capture pooling of eight indexed libraries followed by one pooled hybridization and capture. For the custom design below 3 Mb, the probe volume was applied per hybridization reaction: 2 µL of custom probe was used in each of the eight individual hybridizations in Run 1, and 2 µL was used for the single eight-library pooled hybridization in Run 2. The protocol describes the implemented workflow and does not constitute formal analytical validation.

Reagents and materials

1. **Agilent SureSelect Enzymatic Fragmentation Kit,** catalogue no. 5191-4079; stored at -20 °C.
2. **Agilent SureSelect Library Prep kit,** catalogue no. 5500-0146; stored at -20 °C.
3. **Agilent SureSelect XT HS2 Index Primer Pairs 1-16, single-use,** catalogue no. 5191-5687; stored at -20 °C.
4. **Agilent custom Tier 1 biotinylated RNA probes,** catalogue no. 5191-6900; stored at -80 °C.
5. **Agilent SureSelect Hybridization and Wash Module,** catalogue no. 5190-9685; components stored according to the manufacturer's instructions.
6. **Agilent SureSelect Post-Capture PCR Module,** catalogue no. 5191-6686; stored at -20 °C.
7. **AMPure XP beads, Beckman Coulter,** catalogue nos. A63880/A63881; stored at 2-8 °C; do not freeze.
8. **Dynabeads MyOne Streptavidin T1, Thermo Fisher Scientific,** catalogue no. 65601; stored at 2-8 °C.
9. **Illumina MiSeq Reagent Nano Kit v2, 300 cycles,** used for Run 1; stored and handled according to the manufacturer's instructions.
10. **Illumina MiSeq Reagent Micro Kit v2, 300 cycles,** used for Run 2; stored and handled according to the manufacturer's instructions.

Additional reagents and equipment. 1x Low TE, nuclease-free water, freshly prepared 70% ethanol, absolute ethanol, PCR tubes or plates, filtered tips, calibrated pipettes, magnetic racks, a thermal cycler with heated lid, a vortex mixer, a plate mixer, Qubit fluorometry, and capillary electrophoresis using a Bioanalyzer, TapeStation, or equivalent system. RNase-free handling was applied from hybridization and capture onward; preceding steps were performed under standard clean DNA-library conditions. Master-mix volumes for eight samples include the manufacturer-specified pipetting excess where provided.

Run 1 - Individual capture followed by post-capture pooling

0. Genomic DNA quality control

- Quantify each genomic DNA sample using the Qubit dsDNA High-Sensitivity assay. Assess DNA integrity by TapeStation, Bioanalyzer, or an equivalent method when available.
- Use 10-200 ng genomic DNA per sample in a maximum volume of 7.0 µL. Adjust each sample to 7.0 µL with nuclease-free water or 1x Low TE.

1. Enzymatic fragmentation

- **Kit:** SureSelect Enzymatic Fragmentation Kit (5191-4079).
- **Eight-sample master mix:** 18 µL 5x SureSelect Fragmentation Buffer + 9 µL SureSelect Fragmentation Enzyme = 27 µL.
- Dispense 3 µL master mix into each 7-µL DNA sample to obtain a final volume of 10 µL.
- **Thermal program:** 37 °C for 10 min; 65 °C for 5 min; hold at 4 °C.
- At the 4 °C hold, add 40 µL nuclease-free water to each sample (320 µL total) and proceed directly to end repair/A-tailing.

2. End repair and A-tailing

- **Kit:** SureSelect Library Prep kit (5500-0146).
- **Eight-sample master mix:** 144 µL End Repair-A Tailing Buffer + 36 µL End Repair-A Tailing Enzyme Mix = 180 µL.
- Dispense 20 µL master mix into each 50-µL fragmented-DNA sample to obtain a final volume of 70 µL.
- **Thermal program:** 20 °C for 15 min; 72 °C for 15 min; hold at 4 °C.

3. Adaptor ligation and ligation clean-up

- **Eight-sample ligation master mix:** 207 µL Ligation Buffer + 18 µL T4 DNA Ligase = 225 µL.
- Add 25 µL ligation master mix and 5 µL SureSelect XT HS2 Adaptor Oligo Mix to each 70-µL sample. Final reaction volume: 100 µL.
- **Thermal program:** 20 °C for 30 min; hold at 4 °C.
- **Clean-up:** Add 80 µL AMPure XP beads per sample (640 µL total), incubate for 5 min at room temperature, collect magnetically, and wash twice with 200 µL freshly prepared 70% ethanol per sample (3.2 mL total). Briefly dry the beads, elute in 35 µL nuclease-free water per sample (280 µL total), and transfer approximately 34 µL cleared eluate.

4. Indexed pre-capture PCR and library quality control

- **Kits:** SureSelect Library Prep kit (5500-0146) and SureSelect XT HS2 Index Primer Pairs 1-16 (5191-5687). Dual indexing was performed during this PCR.
- **Eight-sample PCR master mix:** 90 µL 5x Herculase II Buffer with dNTPs + 9 µL Herculase II Fusion DNA Polymerase = 99 µL.
- Add 11 µL master mix and 5 µL of the assigned unique Index Primer Pair to 34 µL purified library per sample. Final reaction volume: 50 µL.
- **PCR program:** 98 °C for 2 min; 8 cycles for 100-200 ng high-quality input DNA, 9 cycles for 50 ng, or 11 cycles for 10 ng (98 °C for 30 s, 60 °C for 30 s, and 72 °C for 1 min); 72 °C for 5 min; hold at 4 °C.
- **Clean-up:** Add 50 µL AMPure XP beads per sample (400 µL total), wash twice with 200 µL 70% ethanol per sample (3.2 mL total), and elute in 15 µL nuclease-free water per sample (120 µL total).
- **Quality control:** Quantify each indexed library and assess fragment-size distribution by capillary electrophoresis. The observed amplified-library distribution was approximately 300-450 bp.

5. Individual hybridization with the custom probe

- **Kits:** custom Tier 1 biotinylated RNA probes (5191-6900) and SureSelect Hybridization and Wash Module (5190-9685).
- Prepare eight separate hybridization reactions. Use 500-1000 ng indexed library per sample, adjust each library to 12 µL with nuclease-free water, and add 5 µL SureSelect XT HS2 Blocker Mix per sample (40 µL total).
- **25% RNase Block solution for eight reactions:** 4.5 µL SureSelect RNase Block + 13.5 µL nuclease-free water = 18 µL.
- **Probe Hybridization Mix for eight reactions and a design below 3 Mb:** 18 µL 25% RNase Block + 18 µL custom probe + 54 µL SureSelect Fast Hybridization Buffer + 27 µL nuclease-free water = 117 µL. Add 13 µL to each reaction; each hybridization therefore receives 2 µL custom probe.
- **Fast-Hyb program:** 95 °C for 5 min; 65 °C for 10 min; 65 °C for 1 min and pause; add the Probe Hybridization Mix; 60 cycles of 65 °C for 1 min and 37 °C for 3 s; brief hold at 65 °C. For a two-day workflow, the final hold may be replaced by 21 °C for up to 16 h.

6. Streptavidin-bead capture and washing

- Use 50 µL Dynabeads MyOne Streptavidin T1 per hybridization reaction (400 µL total).

- Wash the beads three times with 200 µL SureSelect Binding Buffer per reaction and resuspend in 200 µL Binding Buffer; minimum Binding Buffer required for eight reactions: 6.4 mL.
- Transfer each approximately 30-µL hybridization reaction to its corresponding bead suspension and mix for 30 min at room temperature at 1400-1900 rpm.
- Wash once with 200 µL SureSelect Wash Buffer 1 per reaction (1.6 mL total), followed by six washes with 200 µL prewarmed SureSelect Wash Buffer 2 per reaction (9.6 mL total). Incubate each Wash Buffer 2 wash for 5 min at 70 °C.
- After the final wash, resuspend each bead-bound captured library in 25 µL nuclease-free water (200 µL total).

7. Post-capture PCR, final clean-up, and quality control

- **Kit:** SureSelect Post-Capture PCR Module (5191-6686).
- **Eight-reaction master mix:** 117 µL nuclease-free water + 90 µL 5x Herculase II Buffer with dNTPs + 9 µL Herculase II Fusion DNA Polymerase + 9 µL SureSelect Post-Capture Primer Mix = 225 µL.
- Add 25 µL post-capture PCR master mix to each 25-µL bead suspension. Final reaction volume: 50 µL.
- **PCR program:** 98 °C for 2 min; 11-12 amplification cycles (98 °C for 30 s, 60 °C for 30 s, and 72 °C for 1 min); 72 °C for 5 min; hold at 4 °C.
- **Final clean-up:** Collect the streptavidin beads magnetically, transfer approximately 50 µL PCR supernatant to a fresh well, add 50 µL AMPure XP beads per library (400 µL total), wash twice with 200 µL 70% ethanol per library (3.2 mL total), and elute in 25 µL 1x Low TE per library (200 µL total).
- **Quality control:** Quantify each captured library by Qubit and assess fragment-size distribution using a high-sensitivity capillary-electrophoresis assay.

8. Post-capture pooling and MiSeq sequencing

- Normalize the eight captured libraries on the basis of concentration and mean fragment size and combine them in equimolar proportions after final post-capture quality control.
- Denature and dilute the final pool and add PhiX according to the applicable MiSeq v2 run setup.
- Sequence using the Illumina MiSeq Reagent Nano Kit v2, 300 cycles, with paired-end 2 x 150-bp reads and dual indexing.

Run 2 – Study normalized pre-capture pooling followed by pooled capture

0. Genomic DNA quality control

- Quantify each genomic DNA sample using the Qubit dsDNA High-Sensitivity assay. Assess DNA integrity by TapeStation, Bioanalyzer, or an equivalent method when available.
- Use 10-200 ng genomic DNA per sample in a maximum volume of 7.0 µL. Adjust each sample to 7.0 µL with nuclease-free water or 1x Low TE.

1. Enzymatic fragmentation

- **Kit:** SureSelect Enzymatic Fragmentation Kit (5191-4079).
- **Eight-sample master mix:** 18 µL 5x SureSelect Fragmentation Buffer + 9 µL SureSelect Fragmentation Enzyme = 27 µL. Dispense 3 µL into each 7-µL DNA sample.
- **Thermal program:** 37 °C for 10 min; 65 °C for 5 min; hold at 4 °C. At the 4 °C hold, add 40 µL nuclease-free water per sample (320 µL total).

2. End repair and A-tailing

- **Eight-sample master mix:** 144 µL End Repair-A Tailing Buffer + 36 µL End Repair-A Tailing Enzyme Mix = 180 µL. Dispense 20 µL into each 50-µL fragmented-DNA sample.
- **Thermal program:** 20 °C for 15 min; 72 °C for 15 min; hold at 4 °C.

3. Adaptor ligation and ligation clean-up

- **Eight-sample ligation master mix:** 207 µL Ligation Buffer + 18 µL T4 DNA Ligase = 225 µL. Add 25 µL master mix and 5 µL SureSelect XT HS2 Adaptor Oligo Mix to each sample.
- **Thermal program:** 20 °C for 30 min; hold at 4 °C.
- **Clean-up:** Add 80 µL AMPure XP beads per sample (640 µL total), wash twice with 200 µL 70% ethanol per sample (3.2 mL total), elute in 35 µL nuclease-free water per sample (280 µL total), and transfer approximately 34 µL cleared eluate.

4. Indexed pre-capture PCR and library quality control

- **Eight-sample PCR master mix:** 90 µL 5x Herculase II Buffer with dNTPs + 9 µL Herculase II Fusion DNA Polymerase = 99 µL. Add 11 µL master mix and 5 µL unique Index Primer Pair to 34 µL library per sample.
- **PCR program:** 98 °C for 2 min; 8 cycles for 100-200 ng high-quality input DNA, 9 cycles for 50 ng, or 11 cycles for 10 ng (98 °C for 30 s, 60 °C for 30 s, and 72 °C for 1 min); 72 °C for 5 min; hold at 4 °C.
- **Clean-up:** Add 50 µL AMPure XP beads per sample (400 µL total), wash twice with 200 µL 70% ethanol per sample (3.2 mL total), and elute in 15 µL nuclease-free water per sample (120 µL total).
- **Quality control:** Quantify each indexed library and assess fragment-size distribution by capillary electrophoresis. The observed amplified-library distribution was approximately 300-450 bp.

5. Normalized eight-library pre-capture pooling

- Quantify the eight indexed libraries. Calculate the volume of each library required to provide equivalent DNA-mass contributions and combine the eight libraries before hybridization.
- Concentrate the normalized pool to less than 12 µL in a vacuum concentrator at no more than 45 °C without overdrying. Adjust the pooled library to 12 µL with nuclease-free water, mix thoroughly, and keep on ice.

6. Pooled hybridization with the custom probe

- **Kits:** custom Tier 1 biotinylated RNA probes (5191-6900) and SureSelect Hybridization and Wash Module (5190-9685).
- Treat the normalized eight-library pool as one hybridization reaction and add 5 µL SureSelect XT HS2 Blocker Mix to the 12-µL pool.
- **25% RNase Block solution for one pooled reaction:** 0.5 µL SureSelect RNase Block + 1.5 µL nuclease-free water = 2 µL.
- **Probe Hybridization Mix for one pooled reaction and a design below 3 Mb:** 2 µL 25% RNase Block + 2 µL custom probe + 6 µL SureSelect Fast Hybridization Buffer + 3 µL nuclease-free water = 13 µL. Add the complete mix to the eight-library pool. Thus, 2 µL custom probe was used for the single pooled hybridization reaction.
- **Fast-Hyb program:** 95 °C for 5 min; 65 °C for 10 min; 65 °C for 1 min and pause; add the Probe Hybridization Mix; 60 cycles of 65 °C for 1 min and 37 °C for 3 s; brief hold at 65 °C. For a two-day workflow, the final hold may be replaced by 21 °C for up to 16 h.

7. Streptavidin-bead capture and washing of the pooled library

- Use 50 µL Dynabeads MyOne Streptavidin T1 for the pooled reaction.
- Wash the beads three times with 200 µL SureSelect Binding Buffer and resuspend in 200 µL Binding Buffer; minimum Binding Buffer required: 800 µL.

- Transfer the complete approximately 30-µL pooled hybridization reaction to the washed beads and mix for 30 min at room temperature at 1400-1900 rpm.
- Wash once with 200 µL SureSelect Wash Buffer 1, followed by six washes with 200 µL prewarmed SureSelect Wash Buffer 2 (1.2 mL total). Incubate each Wash Buffer 2 wash for 5 min at 70 °C.
- After the final wash, resuspend the bead-bound captured pool in 25 µL nuclease-free water.

8. Post-capture PCR, final clean-up, and quality control

- **Kit:** SureSelect Post-Capture PCR Module (5191-6686).
- **One-reaction PCR master mix:** 13 µL nuclease-free water + 10 µL 5x Herculase II Buffer with dNTPs + 1 µL Herculase II Fusion DNA Polymerase + 1 µL SureSelect Post-Capture Primer Mix = 25 µL.
- Add the complete 25-µL post-capture PCR master mix to the 25-µL bead-bound captured pool. Final reaction volume: 50 µL.
- **PCR program:** 98 °C for 2 min; 14 amplification cycles (98 °C for 30 s, 60 °C for 30 s, and 72 °C for 1 min); 72 °C for 5 min; hold at 4 °C.
- **Final clean-up:** Collect the streptavidin beads magnetically, transfer approximately 50 µL PCR supernatant to a fresh well, add 50 µL AMPure XP beads, wash twice with 200 µL 70% ethanol, and elute in 25 µL 1x Low TE.
- **Quality control:** Quantify the captured pool by Qubit and assess fragment-size distribution using a high-sensitivity capillary-electrophoresis assay. The eight indexed libraries remain in a single captured pool.

9. MiSeq sequencing

- No second pooling step was performed. Denature and dilute the captured pool and add PhiX according to the applicable MiSeq v2 run setup.
- Sequence using the Illumina MiSeq Reagent Micro Kit v2, 300 cycles, with paired-end 2 x 150-bp reads and dual indexing.

Manufacturer documents

1. Agilent Technologies. SureSelect XT HS2 DNA with Post-capture Pooling: Quick Start Protocol. G9983-90500 Rev A0; 2022.

2. Agilent Technologies. SureSelect XT HS2 DNA with Pre-capture Pooling: Quick Start Protocol. G9985-90500 Rev A0; 2022.

3. Agilent Technologies. SureSelect Enzymatic Fragmentation Kit Protocol. G9702-90050.
